# One Step Further: A Flexible Metal–Organic Framework that Functions as a Dual-Purpose Water Vapor Sorbent

**DOI:** 10.1021/acsmaterialslett.4c02019

**Published:** 2025-01-02

**Authors:** Samuel M. Shabangu, Andrey A. Bezrukov, Alan C. Eaby, Sousa Javan Nikkhah, Shaza Darwish, Varvara I. Nikolayenko, Debobroto Sensharma, Shi-Qiang Wang, Matthias Vandichel, Michael J. Zaworotko

**Affiliations:** † Department of Chemical Sciences, Bernal Institute, University of Limerick, Limerick V94 T9PX, Republic of Ireland; ‡ Institute of Materials Research and Engineering, Agency for Science, Technology and Research, 138634, Singapore

## Abstract

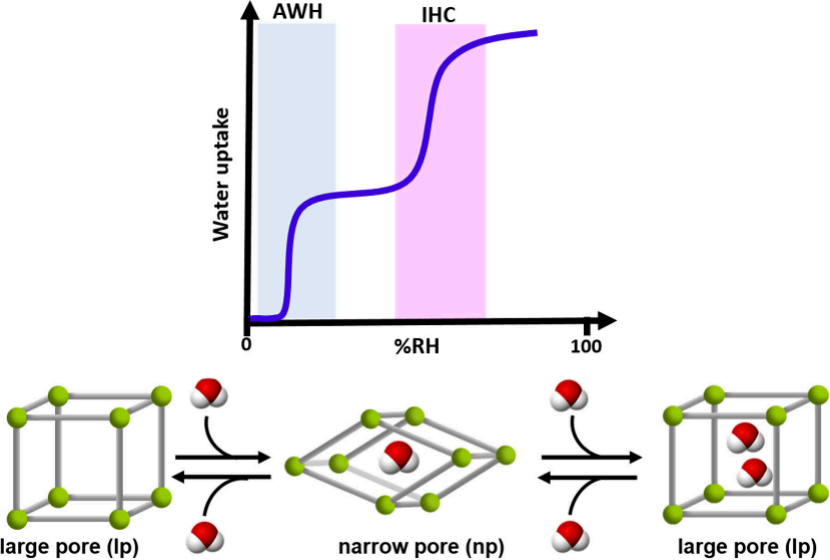

We report a water induced phase transformation in a flexible MOF, [Zn_3_(OH)_2_(btca)_2_] (Hbtca = 1H-benzotriazole-5-carboxylic acid), that exhibits a two-step water vapor sorption isotherm associated with water-induced phase transformations. Variable temperature X-ray diffraction studies revealed that the dehydrated phase, LP-β, is almost isostructural with the previously reported solvated phase, LP-α. LP-β reversibly transformed to a partially hydrated phase, NP, at 5% RH, and a fully hydrated phase, LP-γ, at 47% RH. Structural studies reveal that host–guest and guest–guest interactions are involved in the NP, LP-α, and LP-γ phases. The LP-β phase, however, is atypical; molecular modeling studies indicating that it is indeed energetically favorable as a LP structure. To our knowledge, [Zn_3_(OH)_2_(btca)_2_] is only the second sorbent that exhibits water induced LP-NP-LP transformations (after MIL-53) and represents the first regeneration optimized sorbent (ROS) with two steps at RH ranges relevant for both atmospheric water harvesting and dehumidification.

Population growth and climate change have resulted in water scarcity in many locations.^1^ Arid and semiarid climates are particularly vulnerable as their already limited water supply cannot be replenished quickly enough to meet utility. In low humidity climates, where relative humidity (RH) is typically 10–40%, water scarcity is compounded because traditional water-harvesting technologies such as fogwater collection are rendered inefficient.^2^ Atmospheric water harvesting (AWH) using water vapor sorbents (desiccants) is an emerging technology that could address water scarcity even under low humidity by improving the productivity and energy efficiency of moisture collection.^3^ In a typical AWH process, water vapor from the atmosphere is adsorbed by a sorbent with optimal kinetics and low energy of regeneration, a regeneration optimized sorbent (ROS),^4^ and concentrated after the desorption step by condensation.^5,6^

Another application for sorbent-based technologies is indoor humidity control (IHC).^7,8^ In the absence of adequate IHC measures, prolonged exposure to toxigenic fungi can trigger high levels of allergies and infectious diseases.^9,10^ Consequently, the American Society of Heating, Refrigerating and Air-Conditioning Engineers recommends that the appropriate IHC range for an indoor environment is 45–65% RH.^11^ Unfortunately, current IHC systems have large energy penalties that are the major component of a building’s energy consumption.^11^ Therefore, an ROS suitable for passive IHC in the range of 45–65% RH could reduce, perhaps significantly, the energy footprint of IHC systems. Sorbent-based technologies for water harvesting and humidity control require a suitable ROS which exhibits the following features:^12−16^ (i) high working capacity; (ii) hydrolytically and mechanically robust; (iii) sorbent regeneration at relatively low temperatures (<80 °C for AWH and IHC); and (iv) rapid adsorption and desorption of water.^4,17^ Conventional inorganic desiccants are exemplified by zeolites, silica gel, and hygroscopic salts.^18^ Strongly hydrophilic desiccants like zeolites typically exhibit Type I^19^ sorption isotherms with steep uptake at low RH (Scheme 1a, blue). Moderately hydrophilic desiccants such as silica gels that are optimized for dehumidification, e.g., Syloid AL-1^4^ and Mobil Sorbead R,^20^ tend to exhibit more linear Type I isotherms with less uptake at low RH (Scheme 1a, red). Inorganic salts and mesoporous inorganics such as MCM-41^21^ tend to exhibit stepped or Type V^19^ isotherms, also with lower uptake at low RH (Scheme 1a, green). Most recently, the study of the water sorption properties of metal–organic frameworks, MOFs,^22,23^^24^^25−27^ has resulted in families of rigid desiccants with Type V isotherms that exhibit steps at low RH (Scheme 1a, gold).^28−31^ In the context of AWH, rigid desiccants are exemplified by MOF-303,^32^ UiO-66,^29^ Al-fumarate,^33^ MOF-801,^28^ Co_2_Cl_2_BTDD,^34^ CAU-10(Al)-H,^35^ CAU-23-Al,^36^ Zr-Fumarate,^37^ Cr-soc-MOF-1,^38^ ROS-37,^39,40^ and ROS-39.^41^ Other rigid desiccants perform at higher RH values and so are more suited for passive IHC, as exemplified by Y-shp-MOF-5^11^ and MIL-100(Fe).^7^ Desiccants with low uptake at low RH are unlikely to be suitable for AWH. Further, whereas zeolites are well-suited to adsorb water vapor at very low RH levels, they are poorly suited for AWH or IHC because of the high energy required for desorption.^42,43^ An ideal desiccant for AWH^44^ or passive IHC^38^ would exhibit a water vapor isotherm with a steep humidity-triggered step at <30% or *ca*. 60% RH respectively, with little or no hysteresis for AWH and a desorption branch below 45% RH for passive IHC. Unfortunately, AWH and IHC are usually mutually exclusive in that a desiccant is unlikely to exhibit dual purpose performance parameters.

**Scheme 1:**
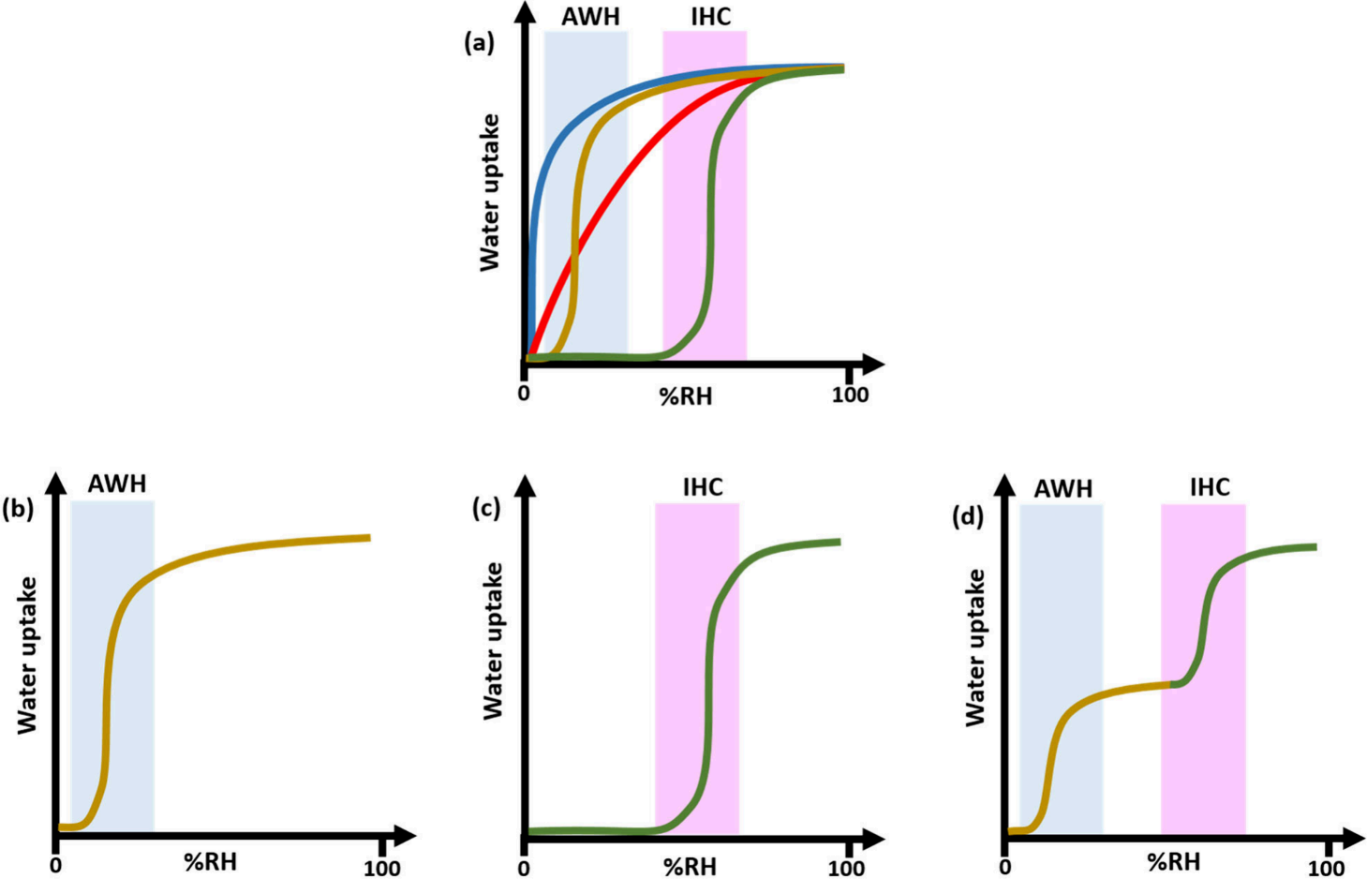
(a) Schematic Illustration of Characteristic Water Vapor Isotherm Shapes of Rigid Desiccants and Characteristic Water Vapor Isotherm Shapes of Flexible Desiccants (b) Type F-IV^S^ (Single Step at Low RH), (c) Type F-IV^S^ (Single Step at Moderate RH), and (d) Type F-IV^m^ (Multistep) Isotherm Type I (strongly hydrophilic, blue); Type I (moderately hydrophilic, red); Type V (step at high RH, green); Type V (step at low RH, gold). The powder blue and pink regions of each diagram are the RH ranges of interest for AWH and IHC, respectively.

Nonrigid or structurally flexible materials can also exhibit stepped isotherms driven by a different mechanism: water-induced phase transformation(s) of nonporous (closed pore, CP) to large pore (LP) transformations^45−47^ can afford single-step type F-IV^S^ isotherms^48^ (F = flexible, S = single step, Scheme 1b (left, middle), and CP to narrow pore (NP) to LP phases can result in Type F-IV^m^ sorption isotherms^49^ (Scheme 1b, right) that, to our knowledge, do not exist in traditional inorganic desiccants. Once again, dual purpose performance is out of reach. Flexible metal–organic materials (FMOMs) with two water-induced phase transformations at appropriate RH values would be ideal candidates to serve as dual-purpose sorbents. Unfortunately, to our knowledge, none have yet been reported. As a part of our interest in the dynamic behavior of porous solids,^48^ we were motivated to investigate the water sorption properties of the previously reported FMOM [Zn_3_(OH)_2_(btca)_2_] (Hbtca = 1H-benzotriazole-5-carboxylic acid), which undergoes several phase transformations induced by gas adsorption/desorption.^50,51^ Herein, we report a detailed investigation of the water sorption properties and dynamic behavior of [Zn_3_(OH)_2_(btca)_2_], which was previously reported to exhibit indications of humidity-induced transformations.^52^ Our study reveals that LP–NP–LP transformations occurred during water uptake, leading to two steps in its water vapor sorption isotherm. Importantly, the two steps are at thresholds suitable for AWH and passive IHC, respectively. Insight into the observed water sorption behavior is provided by single-crystal X-ray diffraction (SCXRD), variable-temperature powder X-ray diffraction (VT-PXRD) and computational experiments.

Single crystals of [Zn_3_(OH)_2_(btca)_2_] were obtained by solvothermal reaction of H_2_btca and Zn(NO_3_)_2_·6H_2_O in *N*,*N*-dimethylformamide (DMF) and H_2_O following a reported procedure.^50^ Single-crystal X-ray diffraction (SCXRD) analysis of the as-synthesized crystals revealed DMF and H_2_O molecules within its pores, in agreement with the previously reported crystal structure.^50^ [Zn_3_(OH)_2_(btca)_2_]·DMF·4H_2_O (herein referred to as LP-α) adopted space group *C*2/*c* with unit cell parameters *a* = 18.5505(7) Å, *b* = 11.6548(5) Å, and *c* = 11.0031(4) Å (Table S1). Bulk phase purity was established using powder X-ray diffraction (PXRD, Figure S1).

Previous reports concerning [Zn_3_(OH)_2_(btca)_2_] studied its gas sorption properties^50−52^ but did not address water-induced phase transformation even though activated crystals exposed to the atmosphere during handling were reported to form a hydrated phase.^52^ We found that immersion of LP-α crystals in H_2_O (3 × 72 h, 323 K) afforded an unreported hydrate [Zn_3_(OH)_2_(btca)_2_]·8H_2_O (herein referred to as LP-γ, Figure 1a) that is isostructural to LP-α, with a similar PXRD pattern to LP-α. (Figure S1). Four symmetry independent H_2_O molecules (O1W–O4W, see ESI for details) disordered over two positions (major component A and minor component B) occupy the cavities that have a pore limiting diameter *d*_lim_ = 4.9 Å and maximum cavity diameter *d*_max_ = 5.9 Å. The water molecules of hydration form a hydrogen-bonded chain with the O_H2O_···O_H2O_ distances ranging from 2.439 to 3.004 Å (Table S2). Three of the four symmetry independent water molecules (O1W–O3W) form H-bonds with either the bridging hydroxo (O1WA···O3 = 2.830 Å) or carboxyl groups (O2WA···O14 = 3.010 Å, O3WA···O15 = 3.183 Å) of the host framework. Thermogravimetric analysis (TGA, Figure S2) of LP-γ revealed two mass loss event of 12 wt % (4 molecules/formula unit) with onset temperatures of 311 and 348 K, respectively. Single crystallinity was retained during heating, allowing for structure determination by SCXRD, which revealed that the first water loss at 311 K afforded the previously reported tetrahydrate,[Zn_3_(OH)_2_(btca)_2_]·4H_2_O (hereinafter referred to as NP, Figure 1b).

**Figure 1:**
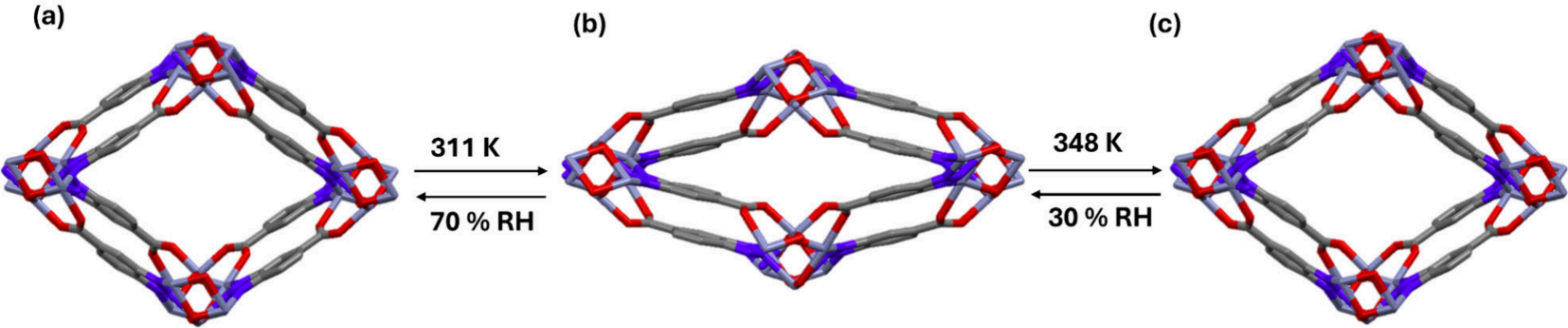
Reversible LP-NP-LP water-induced structural transformations exhibited by [Zn_3_(OH)_2_(btca)_2_]: (a) LP-γ, (b) NP, and (c) LP-β.

The single-crystal-to-single-crystal (SC–SC) transformation from LP-γ to NP resulted in contraction along the crystallographic *b*-axis with concomitant expansion along the *a*-axis (*d*_lim_ = 2.77 Å and *d*_max_ = 3.50 Å, Table S1). A different PXRD pattern with strong peaks shifted to higher 2θ values being observed, indicating a structural transformation to a smaller unit cell (Figure.S1). This phase transformation was also studied in a powdered sample by using VT-PXRD (Figure 2). Water molecules in NP H-bond with the host and the other water molecules (Table S3): hydroxo (O1WA···O3 = 2.740 Å) and carboxyl groups (O2WA···O14 = 2.839 Å, O2WA···O15 = 3.073 Å) and water–water interactions (O1WA··· O2WA = 2.741 Å, O2WA··· O2WA = 3.133 Å); further hydrogen bond interactions are summarized in Figure S3. Heating an NP crystal on the SCXRD goniometer at 373 K resulted in transformation to the anhydrate phase LP-β. The NP → LP-β phase transformation resulted in expansion along the crystallographic *a*-axis with concomitant contraction along the *b*-axis (*d*_lim_ = 5.29 Å and *d*_max_ = 6.53 Å, Table S1). The absence of electron density in the difference electron density maps (Figure S4) and TGA indicate that LP-β is guest-free. Notably, the reverse transformation occurred within minutes under ambient conditions (ca. 50% RH, 298 K), indicating reversibility. Such LP–NP–LP transformations are counterintuitive and rare in FMOMs, MIL-53 being the prototypal example.^53,54^ As discussed below, this phenomenon can be attributed to an induced fit mechanism of water binding.^55,56^

**Figure 2:**
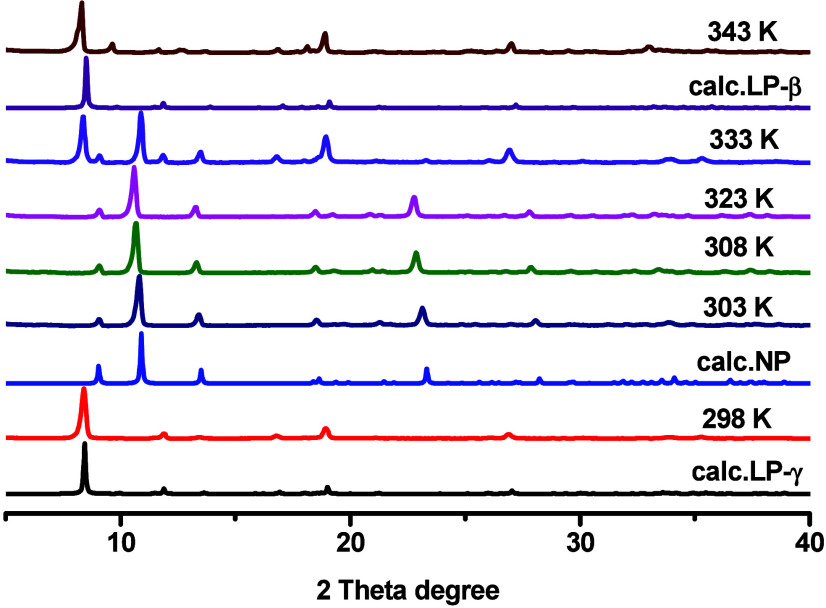
VT-PXRD diffractograms of [Zn_3_(OH)_2_(btca)_2_] collected under air.

To gain additional insight into the water induced phase transformations of LP-β, NP, and LP-γ, Fourier transform infrared (FTIR) spectroscopy experiments were conducted. The anhydrous phase, LP-β, was prepared by heating the as-synthesized powdered sample of [Zn_3_(OH)_2_(btca)_2_] at 373 K under a dynamic vacuum (1 mbar) for 12 h and kept under dry N_2_ to prevent exposure to moisture during handling and data collection. The absence of broad peaks in the range 3700–2700 cm^–1^ of the FTIR spectrum of LP-β (Figure S5a) indicate dehydration,^57^ whereas the sharp, low-intensity peak at 3654 cm^–1^ corresponds to the bridging hydroxyl group (μ_2_-OH).^57,58^ If nanoconfined water had been present then sharp peaks would have been expected.^59^ The hydrous NP phase was studied under a laboratory atmosphere (44% RH and 293 K measured using a diagnostic psychrometer), conditions that are consistent with its generation (Figure 3). After 1 min of exposure, a broad peak centered at 3391 cm^–1^ appeared, consistent with water adsorption.^57^ The relative peak intensity increased at 2 min, and no further changes were observed after 3 and 20 min of exposure, indicating that adsorption to the NP phase was complete within 2 min. This rapid uptake is relatively fast and in line with the inflection in the isotherm (Figure 3). Another difference between the FTIR spectra of the LP-β and NP phases was the presence of a peak at 743 cm^–1^ in LP-β corresponding to a C_aromatic_–H bending frequency from the btca ligand. This peak disappeared during hydration, perhaps because of the changed environment of the C_aromatic_–H moieties following water adsorption. A peak at 1548 cm^–1^ in the LP-β spectrum and 1545 cm^–1^ in the NP spectrum is attributed to the deprotonated (O–C–O)^−^ moiety from the btca ligand (Figure S5b).

**Figure 3:**
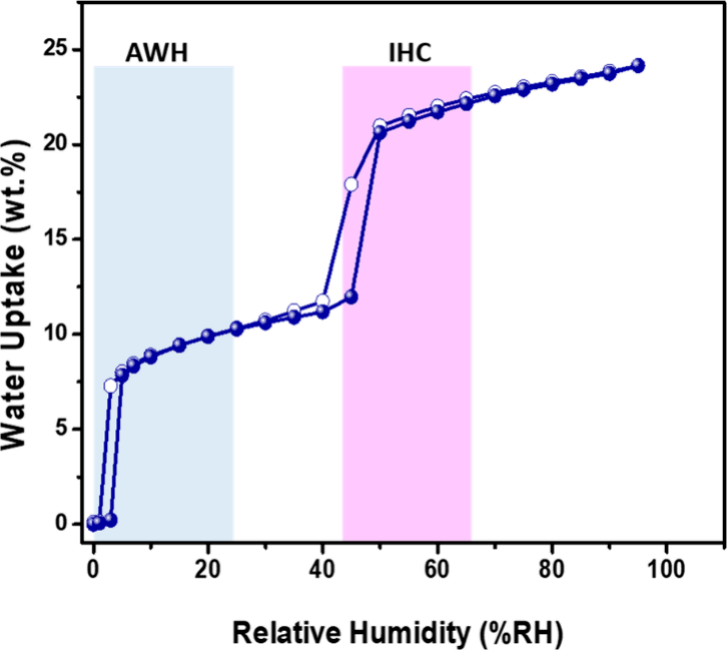
Water vapor sorption isotherm collected (300 K) by dynamic vapor sorption (DVS).

LP-γ was prepared by storage in a desiccator at 84% RH (KCl salt solution, 293 K) for 1 h. The most notable difference in the IR spectrum is an increase in the relative peak intensity of water^60^ and a slight shift in this peak with respect to that of NP (3381 cm^–1^ vs 3374 cm^–1^). No other significant peak shifts during the water vapor induced phase changes were noted.

To further investigate the water sorption properties of [Zn_3_(OH)_2_(btca)_2_], dynamic water vapor sorption (DVS) experiments were conducted after activating a microcrystalline sample of LP-γ under dry air flow at 373 K, thereby inducing the transformation to LP-β. The water vapor sorption isotherm (Figure 3) was collected at 300 K and contains two steps at 5% and 47% RH with uptakes of *ca*. 7.8 and 20.6 wt %, respectively, and the maximum water uptake was observed to be 24.5 wt % at 95% RH. These uptakes are consistent with the TGA and SCXRD data. The isotherm collected at 333 K revealed a shift of the inflection point to RH 8% and 52% RH, respectively (Figure S6). The profile of the water sorption isotherm corresponds to a Type F-IV^m^ isotherm,^49^ but the LP–NP–LP mechanism does not involve the characteristic closed pore (CP) to NP to LP transitions typical of a material that undergoes two phase transformations.^49^ Interestingly, desorption revealed little hysteresis and the release of adsorbed water molecules at 0% RH without heating. Hydrolytic stability tests conducted upon a powdered sample of [Zn_3_(OH)_2_(btca)_2_] subjected to 100 hydration–dehydration cycles between 0 and 60% RH at 300 K revealed retention of water sorption capacity (Figure S7) and exhibit hydrolytic stability as evidenced by PXRD before and after the cycling experiment (Figure.S8). [Zn_3_(OH)_2_(btca)_2_] therefore meets the criteria as a dual purpose ROS for AWH and passive IHC with inflection points at appropriate RH ranges suitable for both technologies. Our review of the literature revealed only 7 MOFs with multistep water vapor isotherms: MIL-100(Fe),^31^ MIL-101 (Cr),^61^ MIL-101-NH_2_,^61^ MIL-101-SO_3_H,^61^ [Mn(imH)]_2_[Mo(CN)_8_],^62^ [Fe^II^(prentrz)_2_Pd^II^(CN)_4_],^63^ and MIL-53(Cr),^53,54^ (see Table S4). Out of these, only [Mn(imH)]_2_[Mo(CN)_8_], [FeII(prentrz)_2_PdII(CN)_4_], and MIL-53(Cr) are driven by water-induced structural transformations. Specifically, a Prussian Blue analogue [Mn(imH)]_2_[Mo(CN)_8_] underwent a breathing mechanism due to water inclusion, a Hofmann structure [Fe^II^(prentrz)_2_ Pd^II^(CN)_4_] transformed through a flexible ligand and layer sliding, whereas MIL-53(Cr) underwent induced fit.^58,64−66^ MIL-53(Cr) is an outlier in that its guest-free phase is LP, contracting upon water loading to an NP phase through an induced fit mechanism,^54^ before expanding to the same LP structure at higher RH. These existing MOFs with multistep water sorption isotherms are unsuited for dual purpose AWH and passive IHC because the threshold RH values for their steps meet the criteria for only one application (see Table S4).

Rates of water adsorption/desorption are key performance parameters with respect to assessing utility in water harvesting.^4,32,67,68^ For FMOMs, water vapor sorption kinetics are rarely reported.^60,69−72^ Our group has recently developed and reported an isotherm-based kinetics model^4^ that links water vapor sorption thermodynamics with kinetics and explains differences in sorption kinetics for various sorbents, including structurally rigid sorbents such as ROS-037,^39^ ROS-039,^41^ ROS-040,^73^ MOF-303,^67^ MIL-160,^74^ CAU-10-H,^75^ and Al-fumarate^76^ as well as flexible sorbents such as X-dia-2-Cd.^69^ Interestingly, for 2-stepped isotherms as exhibited by [Zn_3_(OH)_2_(btca)_2_], the model^4^ was found to predict two distinct rates of adsorption, as seen for the adsorption kinetics of MIL-101(Cr)^77^ and the desorption kinetics of [[Mn(imH)]_2_[Mo(CN)_8_]]_n_.^62^ Indeed, humidity swing experiments were conducted on [Zn_3_(OH)_2_(btca)_2_] from 0 to 60% RH at 298 K (see Figure S9), and we observed two distinct water vapor sorption rates (Figure 4a). During the adsorption phase, there was initial fast loading followed by slower adsorption loading. Similarly, during desorption, we observed fast unloading followed by slower unloading. These kinetic profiles were fitted using our isotherm-based kinetics model (Figure 4b), further supporting the validity of the model.^4^ In a separate RH-swing experiment conducted from 0 to 30% RH at 298 K, corresponding to the low RH step in the [Zn_3_(OH)_2_(btca)_2_] isotherm, the kinetics exhibited a constant rate for both adsorption and desorption. For a 5.6 mg sample, adsorption was complete within 15 min, while desorption required 80 min (Figure 4c). This experimental kinetics data is also consistent with our kinetics model (Figure 4d).^4^ With regards to the sorption mechanism, sorption kinetics tends to be limited by diffusion of water vapor to the sorption bed.^4^ Therefore, the position and profile of the inflection are the key factors that impact adsorption kinetics rather than whether adsorption occurs by pore condensation or structural transformation(s).^78^

**Figure 4:**
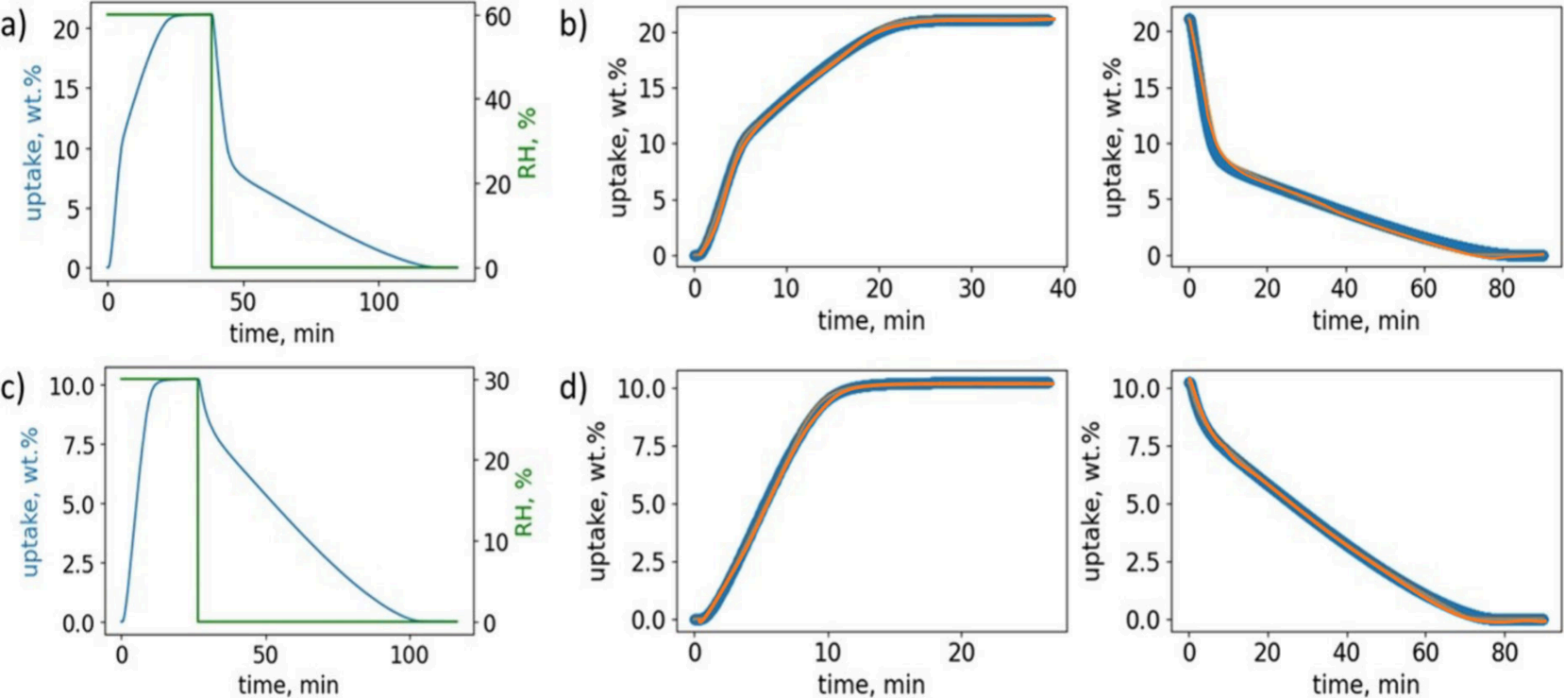
Above: (a) 0–60% RH humidity swing kinetics data collected at 300 K on 5.6 mg of [Zn_3_(OH)_2_(btca)_2_]; (b) 0–60% RH humidity swing kinetics observed (blue) vs calculated using an isotherm-based kinetics model^4^ (orange); Below: (c) 0–30% RH humidity swing kinetics data collected at 300 K on 5.6 mg of [Zn_3_(OH)_2_(btca)_2_]; (d) 0–30% RH humidity swing kinetics observed (blue) vs calculated using the same model^4^ (orange).

The LP–NP–LP mechanism can be rationalized by evaluating the level of distortion of the coordination environment of the metal during the structural transformations that occurred during hydration. During the transformation from LP-β → NP → LP-γ, the relative positions of the Zn and hydroxo atoms in the rod building block that is the backbone of the network structure remained relatively unchanged despite considerable contraction and subsequent expansion along the *b* axis (Figure S10). Instead, the framework flexibility is enabled by changes in the carboxyl and triazolate coordination geometries. The extent of bond distortion can be assessed by structural changes in the framework, specifically the angles δ_1_ (centroid c1–N5–Zn1, Figure S10c) and δ_2_ (centroid c2–centroid c3–C10, Figure S10d). These ligating moieties tend to form coordination bonds that have δ_1_ and δ_2_ values near 180°. The most obtuse angles were observed for LP-β with δ_1_ = 166.22° and δ_2_ = 164.79° whereas NP exhibited more acute angles, δ_1_ = 150.73° and δ_2_ = 141.69°, an indication of strain. However, the existence of this phase indicates that the energy gained by the H-bonded network of water molecules can overcome the geometric strain of the Zn–N_triazolate_ and Zn–O_carboxyl_ coordination bonds. Such distortions were also observed for the LP–NP transformation in MIL-53(Cr) during water loading.^55^ Further water uptake from NP to LP-γ relieves this distortion, with δ_1_ = 162.84° and δ_2_ = 154.31°. This analysis suggests that both NP and LP-γ distort to adapt to the optimal geometry of the H-bond network through an induced fit mechanism. Overall, water adsorption by LP-β resulted in a phase transformation that involved contraction by 24% of the unit cell volume to form NP. This compares to the unit cell volume reduction of 45% in MIL-53. It is reasonable to assert that the induced fit NP–LP transformation can be attributed to water–pore wall interactions that overcome the inherently strained structure of NP. There are also examples of torsional freedom in a linker ligand resulting in enhanced host–guest interactions through a different induced fit mechanism.^79^

To further evaluate the asserted induced fit mechanism and resultant water sorption isotherm profile of [Zn_3_(OH)_2_(btca)_2_], we performed density functional theory (DFT) calculations and grand canonical Monte Carlo (GCMC) simulations. Different crystal structures (structures 0–13) were selected from DFT minimum energy pathways of the empty host material between unit cell volumes ranging incrementally from 1939.72 to 2669.51 Å^3^ (see Table S6 and ESI for details). Adsorption isotherms were calculated for each of the 14 structures using GCMC simulations (Figure S13). Type I^80^ adsorption isotherms were observed for structures 0 to 6 with unit cell volumes between 1939.72 and 2281.735 Å^3^ (Table S6); this profile is characteristic of a unimolecular layer within the pore.^80^ By further increasing the unit cell volume from 2334.673 to 2669.54 Å^3^ (structures 7 to 13, Table S6), the profile of the simulated isotherms became S-shaped (Figure S13). Using the simulated isotherms in Figure S13, a contour plot representing each simulated structure (unit cell volume) with respect to changing RH was constructed (Figure 5). This plot was used to identify the phase transformation landscape of [Zn_3_(OH)_2_(btca)_2_] during water loading. For each RH value in the experimental isotherm, the most probable structure was determined by correlating experimental uptake with the uptake from the simulated isotherms. Starting with empty LP-β at 0% RH (structure 8), the uptake at first loading (4.03% RH) corresponds to structure 3 with a similar cell volume to NP. Further loading (6.12% RH) results in a small cell volume expansion and correlates to structure 4. This phase is maintained until 30.16% RH. Upon further increase in RH, the unit cell volume expands corresponding to most probable structures 5, 9, and 11 at RH values of 36–41.5%, 45.12%, and 51.02% RH, respectively. LP-γ (structure 11) is the predominant phase for >51.02% RH.

**Figure 5:**
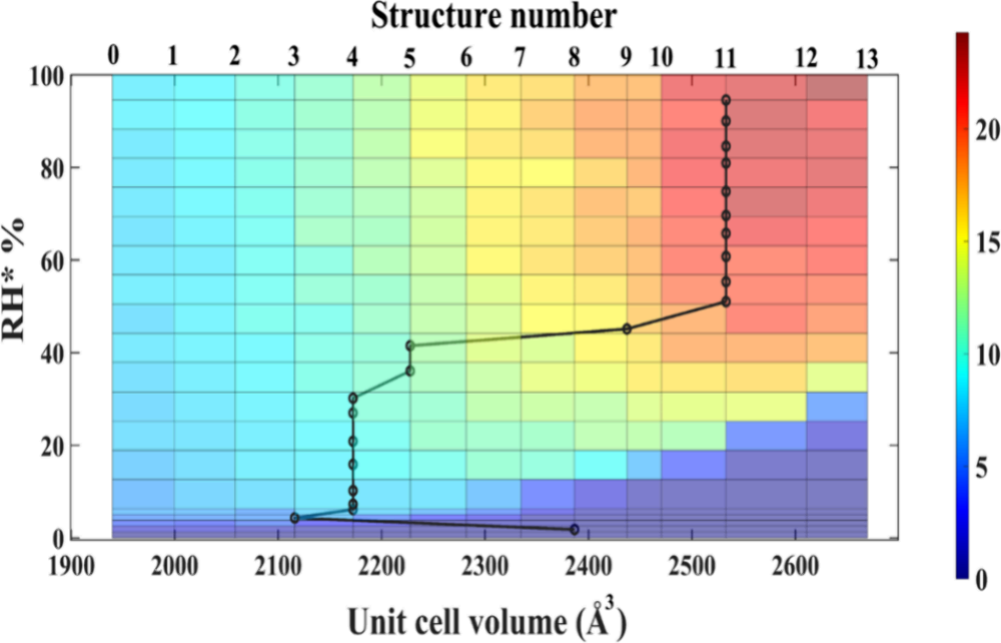
Contour plot representing GCMC simulated H_2_O adsorption isotherms (uptake is color coded) for each of the 14 crystal structures determined from DFT calculations. The black circles/lines represent the unit cell volume of the most probable structure at the specified experimental uptake and RH.

The DFT calculations indicate that the relative energy of the LP-β (structure 10) is −45.59 kJ/mol lower than that of NP (structure 0) which we can attribute to the strain in NP discussed above. Analysis of the experimental and simulated crystal structures thereby allows us to assert that the energy gained from hydrogen bonding interactions between water molecules and the framework of NP can indeed overcome the inherently strained structure of NP. Further loading of water molecules enables LP-γ to be energetically favored over NP above *ca*. 50% RH. The average adsorption energy at the DFT (BEEF-vdW) level stays within the range from −60 to −50 kJ/mol per water during the loading of [Zn_3_(OH)_2_(btca)_2_] (Table S7), with hydrogen bond networks visualized for four relevant water loadings in Figure S14. Furthermore, an energy decomposition analysis of the adsorption energies shows that water–host interactions are dominant at low water loading (low unit cell volumes) and are being surpassed by water–water interactions at higher water loadings in LP phases.

In conclusion, we report the water-induced structural transformations of [Zn_3_(OH)_2_(btca)_2_], an FMOM that underwent atypical LP–NP–LP structural transformations triggered by increasing RH. To our knowledge, this is only the second example of such a two-step transformation after MIL-53. Our work offers two main take home messages. First, with regards to design of FMOMs, induced fit behavior can be governed by at least two mechanisms: torsional freedom in a linker ligand, as seen in sql-SIFSIX-bpe-Zn, can enable a more strained NP phase that is stabilized by enhanced host–guest binding; here we reveal that strain in an NP phase of a sorbent with relatively rigid ligands and RBBs can be overcome by a combination of guest–guest and host–guest interactions, also resulting in induced fit. Second, this work further points toward the potential utility of FMOMs in water harvesting and dehumidification applications, which is much less recognized than for rigid sorbents. Indeed, to our knowledge the first example of an FMOM that offers relatively fast kinetics, hydrolytic stability, small hysteresis, and a step at low RH was only reported in 2023 by us.^69^ This study goes “one step further” than our previous work by detailing the first example of a water sorbent of any type that exhibits a 2-step water sorption isotherm that meets the performance criteria needed to serve as a dual purpose water vapor sorbent thanks to steps suitable for both AWH (5% RH) and passive IHC (47% RH). Nevertheless, there is no expectation that a two-step sorbent such as [Zn_3_(OH)_2_(btca)_2_] will offer better performance for either AWH or IHC when compared to a single-step sorbent with a step at an appropriate threshold. This is because the low RH step would not be in play under typical IHC conditions whereas the intermediate RH step should not occur under AWH (low RH) conditions.

## Data Availability

CCDC 2328383-2328385 contain the supplementary crystallographic data for this work. These data can be obtained free of charge via www.ccdc.cam.ac.uk/data_request/cif, or by emailing data_request@ccdc.cam.ac.uk, or by contacting The Cambridge Crystallographic Data Centre, 12 Union Road, Cambridge CB2 1EZ, UK; fax: + 44 1223 336033.

## Supporting Information Available

Details of synthetic procedures and analytical testing methods, additional crystallographic parameters and analysis, supporting figures, details of computational methodology, and supporting references (PDF)
