# Supplementary material for: One Step Further: A Flexible Metal–Organic Framework that Functions as a Dual-Purpose Water Vapor Sorbent
Source: ACS Mater Lett. 2025 Jan 2;7(2):433–41. doi: 10.1021/acsmaterialslett.4c02019 (PMC11795622; doi:10.1021/acsmaterialslett.4c02019)
Supplement: Supplementary file 1 — tz4c02019_si_001.pdf [file tz4c02019_si_001.pdf]

Supporting Information (SI)

One Step Further: A Flexible Metal-Organic Framework That Functions as a Dual-Purpose Water Vapor Sorbent

Samuel M. Shabangu[a], Andrey A. Bezrukov[a], Alan C. Eaby[a], Sousa Javan Nikkhah[a], Shaza Darwish[a], Varvara I. Nikolayenko[a], Debobroto Sensharma[a], Shi-Qiang Wang[a,b], Matthias Vandichel[a] and Michael J. Zaworotko[a]

[a]Department of Chemical Sciences, Bernal Institute, University of Limerick, Limerick V94 T9PX, Republic of Ireland

[b]Institute of Materials Research and Engineering, Agency for Science, Technology and Research, 138634, Singapore

Table of contents

Methods..5

S1.Experimental……………………………………………………………...…..……...5

S2.Powder X-ray Diffraction (PXRD..5

S3. Thermogravimetric Analyses (TGA).………………………………………..….……....5

S4. Variable-Temperature Powder X-ray Diffraction (VT-PXRD) Measurements....................5

S5. Single-crystal X-ray Diffraction Measurements...5

S6. Fourier Transform Infrared (FTIR) spectroscopy………………………………………..6

S7. Adventure Dynamic Vapor Sorption (DVS)Measurements……………………….....…...6

S8. Gas sorption measurement…………..……………………………………….…………7

S9. Analysis of previous guest-induced transformations in $[Zn_3(OH)_2(btca)_2]$ for hydrated and DMF included crystal structures from the Cambridge Structural Database…………………………..….7

Tables and Figures……………………………………………………...……………..7

References…………………………………………………………………………………25

Table of Figures

Figure S1: Comparison of calculated and experimental PXRD diffractograms of $[Zn_3(OH)_2(btca)_2]$……………………………………………………………………………......8

Figure S2: TGA profiles of $[Zn_3(OH)_2(btca)_2]$ ·DMF·4H_2O and $[Zn_3(OH)_2(btca)_2]$ ·4H_2O..10

Figure S3: hydrogen bond structure between water molecules in the channels of LP-*α* and NP…...11

Figure S4: Electron density maps and crystal structure data of LP-γ ($[Zn_3(OH)_2(btca)_2]$ ·4H_2O), NP ($[Zn_3(OH)_2(btca)_2]$ ·2H_2O) and LP-β ($[Zn_3(OH)_2(btca)_2]$)…………………………………………………………………….………..12

Figure S5: Fourier transform infrared spectroscopy (FT-IR) spectra measured for LP-β, NP and LP-γ showing the (a) full range and (b) the fingerprint region. The time values indicate the time elapsed after first exposure…………………………………………………………………………...……..12

Figure S6: Water vapor sorption isotherm of $[Zn_3(OH)_2(btca)_2]$ collected using an adventure dynamic vapor sorption (DVS) instrument at 333K ……………………………………………...……..13

Figure S7: Water vapor humidity swing cycling of $[Zn_3(OH)_2(btca)_2]$ (0% RH to 60% RH)………………………………………………………...……………………………......13

Figure S8: Comparison of PXRD pattern before and after cycling...………...…...………….............14

Figure S9: Fit of kinetics data (blue) at 300K on $[Zn_3(OH)_2(btca)_2]$ using isotherm-based kinetics model………………………………………………………………………………………………16

Figure S10: Structural overlays of LP-γ (blue) and NP…………………...…...………….............17

Figure S11: CO_2 adsorption measured at 195K……………………………..……….……………18

Figure S12. Water molecule model with atomic point charges according to the Qeq method………………………………………………………………………………………......22

Figure S13. Rescaled simulated adsorption isotherms of H_2O at different unit cell volumes……………………………………………………………………………………………..23

Figure S14. Visualization of hydrogen bonds (presented in dashed blue lines) for selected structures from **Table S7**, (a) 03, (b) 04, (c) 09, and (d) 11……………………………………......................24

Figure S15. 3-dimensional H_2O adsorption isotherms taken from ………….....................................25

Table of Tables

Table S1: Selected crystallographic data for three forms of [$Zn_3(OH)_2(btca)_2$]…………………...8

Table S2: Possible hydrogen bonds involving water molecules modelled for LP-γ …………......9

Table S3: Possible hydrogen bonds involving water molecules modelled for **NP**………………...9

Table S4: Reported multistep water vapor sorption isotherms and their potential application based on their inflection points……………………………………………………….……………………..……..15

Table S5: Reported [$Zn_3(OH)_2(btca)_2$] analogues with their guest molecules and their published sorption properties………………………………………………………………………..………19

Table S6: Cell parameters of structures employed in NEB-run 1 and 2, and the 14 selected structures for further water sorption studies (labeled 00, 01, …, 13)………………………………….………...21

Table S7: Water adsorption energy per water (E_{ads}) as function of loading in 1x1x2 supercells determined after optimization……………………………………………………………..........23

S1.Experimental

Single crystals of $[Zn_3(OH)_2(btca)_2]$ were obtained by the solvothermal reaction of H_2btca and $Zn(NO_3)_2{\cdot}6H_2O$ in *N*,*N*-dimethylformamide (DMF) and H_2O following a reported procedure.[1] A mixture of $Zn(NO_3)_2{\cdot}6H_2O$ (0.1 mmol, 24.9 mg), H_2btca (0.05 mmol, 8.0 mg), DMF (3.0 mL),and H_2O (1.0 mL) was placed into a Pyrex glass vial and heated to 140°C for three days. After cooled to room temperature, the block-shaped yellowish crystals were collected and washed with DMF three times. Solvent exchange of $[Zn_3(OH)_2(btca)_2]{\cdot}DMF{\cdot}4H_2O$ with H_2O (72 hours, 323K) resulted in formation of an unreported hydrate $[Zn_3(OH)_2(btca)_2]{\cdot}8H_2O$.

S2. Powder X-ray Diffraction (PXRD)

Diffractograms were recorded using a PANalytical Empyrean™ diffractometer equipped with a PIXcel3D detector operating in scanning line detector mode with an active length of 4 utilizing 255 channels. The diffractometer is outfitted with an Empyrean Cu LFF (long fine-focus) HR (9430 033 7310x) tube operated at 40 kV and 40 mA and CuKα radiation (λα = 1.540598 Å) was used for diffraction experiments. Continuous scanning mode with the goniometer in the theta-theta orientation was used to collect the data. Incident beam optics included the Fixed Divergences slit with anti-scatter slit PreFIX module, with a 1/8° divergence slit and a 1/4° anti-scatter slit, as well as a 10 mm fixed incident beam mask and a Soller slit (0.04 rad). Divergent beam optics included a P7.5 anti-scatter slit, a Soller slit (0.04 rad) and a Ni-β filter. In a typical experiment, 25 mg of sample was dried, ground into a fine powder and was loaded on a zero background silicon disks. The data was collected from 5°−40° (2θ) with a step-size of 0.0131303° and a scan time of 30 seconds per step. Crude data were analyzed using the X'Pert HighScore Plus™ software V 4.1 (PANalytical, The Netherlands).

S3. Thermogravimetric analysis (TGA)

Thermogravimetric analyses (TGA) were performed under N2 using a TA Instruments Q50 system. Samples were loaded into aluminium sample pans and heated at 5 K min^{-1} from room temperature to 773K.

S4. Variable Temperature Powder X-ray Diffraction (VT-PXRD)

Diffractograms at different temperature were recorded using a PANalytical X'Pert Pro-MPD diffractometer equipped with a PIXcel3D detector operating in scanning line detector mode with an active length of 4 utilizing 255 channels. Anton Paar TTK 450 stage coupled with the Anton Paar TCU 110 Temperature Control Unit was used to record the variable temperature diffractograms. The diffractometer is outfitted with an Empyrean Cu LFF (long fine-focus) HR (9430 033 7300x) tube operated at 40 kV and 40 mA and CuKα radiation (λα = 1.54056 Å) was used for diffraction experiments. Continuous scanning mode with the goniometer in the theta-theta orientation was used to collect the data. Incident beam optics included the Fixed Divergences slit, with a 1/4° divergence slit and a Soller slit (0.04 rad). Divergent beam optics included a P7.5.

S5. Single Crystal X-ray Diffraction.

High quality single crystals of LP-β ($[Zn_3(OH)_2(btca)_2]$), NP ($[Zn_3(OH)_2(btca)_2]{\cdot}4H_2O$) and LP-γ ($[Zn_3(OH)_2(btca)_2]{\cdot}8H_2O$) were chosen for single crystal X-ray diffraction measurements. Diffraction data for LP-β (298 K), NP (100 K) and LP-γ (100 K) were collected on a Bruker Quest diffractometer equipped with a IμS microfocus X-ray source (Cu Kα, λ = 1.54178 Å; Mo Kα, (λ = 0.71073 Å) and CMOS detector. In all cases, data was indexed, integrated and scaled using Bruker SAINT software.[2] Absorption correction was performed by multi-scan method using in SADABS.[3] Space group determination was performed simultaneously with structure solution using SHELXT[3] intrinsic phasing methods through the X-Seed[4,5] graphical user interface. Zinc, carbon, nitrogen, oxygen and hydroxo

hydrogen atoms of the host were refined anisotropically using SHELXL, using full-matrix least squares minimization.[4] Host hydrogen atomic positions were calculated using riding models. H_2O oxygen atoms located in the pores of NP and LP-γ were found to be disordered and these were refined aniostropically with their combined site-occupancy factor constrained to unity. These oxygen atoms were labelled O*n*W*X*, where n = 1–4 and X = A or B for the major component and minor component, respectively. Water hydrogen atoms could not be modelled owing to disorder. Selected crystallographic parameters are reported in Table S2. The O···O distances that correspond to possible hydrogen bond interactions are listed in Tables S4 and S5. Pore volumes and geometries (pore limiting diameters and maximum pore diameters) were calculated using the Pore Analyzer (default settings) feature in Mercury.[5] Crystal structures and void volumes were visualized using Mercury.[5] Difference electron density maps were calculated using OLEX2.[6]

S6. Fourier Transform Infrared (FTIR) spectroscopy

Spectra were obtained by using a FTIR spectrometer (Agilent technologies, Cary 630) in the range of wavelength 4000-650 cm^{-1}. Heating the as-synthesized powdered sample of $[Zn_3(OH)_2(btca)_2]$ to 373 K under dynamic vacuum (1 mbar) for 12 h, afforded FTIR of LP-β. Multiple time dependent measurements were carried out (1 min, 2 min, 3 min and 20 min) by exposure to ambient air at 44 %RH affording NP data. FTIR data for LP-γ was collected after exposure to 84 %RH for 1h.

S7. Water Vapor Sorption Measurements

Water vapor sorption isotherm measurements were performed using Adventure dynamic vapor sorption (DVS) instrument manufactured by Surface Measurement Systems. The instrument gravimetrically measures water vapor uptake using air as a carrier gas. Digital mass flow controllers regulate flows of dry and saturated gases. Relative humidity is generated by precisely mixing dry and saturated gas flows in desired flow ratios which produce expected relative humidity. Pure water was used to generate water vapor for these measurements and temperature was maintained at 298 K or at 333 K by enclosing the system in a temperature-controlled incubator. The mass of the sample was determined by a high-resolution microbalance with a precision of 0.01 μg. Microbalance has symmetric configuration with two branches of the balance being exposed to the same gas and being kept at the same temperature, which allows negation of buoyancy and drag effects. Instrument allows measurement of 2 samples in parallel. Prior to the measurement, sample was in-situ activated in dry air at 393K using built-in preheater and consequently cooled to sorption temperature. Isotherm measurements were performed on approximately 11 mg of sample powder. 400 sccm min^{-1} total flow was used for the measurements at 300 K and 50 sccm min^{-1} total flow was used for measurements at 333 K. The flow is split between two samples. For each isotherm point, dm/dt < 0.01 %/min was used as criteria of reaching equilibrium.

Water vapor sorption cycling was performed at 300 K on a Surface Measurement Systems DVS Intrinsic instrument using air as a carrier gas to gravimetrically measure the uptake and loss of vapor. The mass of the sample was determined by comparison to an empty reference pan and recorded by a high-resolution microbalance with a precision of 0.1 μg. Prior to the measurement, the sample (5.6 mg) was ex-situ activated at 393 K and in situ activated in dry air at 313 K for 60 minutes. Humidity swing was measured between two points 0 and 60 % RH. 100 cycles were subsequently performed, each cycle consisting of 30 min adsorption step (60 % RH) and 80 min desorption step (0 % RH).

Experimental sorption kinetics was measured on DVS Intrinsic instrument using humidity swing experiment performed between two points for 0-30 and 0-60 % RH on a 5.6 mg sample. Experimental sorption kinetics was modelled using isotherm-based kinetics model recently published by us.[7] Adsorption and desorption kinetics was modelled using Eqn. 2. RH_{bed} was determined from adsorption

branch of the isotherm at the corresponding uptake. Two parameters were fitted: k and t0, where t0 was fitted in 0-3 minutes range, fitting parameters could be found in Figure S9.

$$\frac{dw}{dt} = k \cdot (RH_{flow} - RH_{bed}) \qquad \text{Eqn.2}$$

where w is uptake (wt.%), k is kinetics coefficient, RH_{flow} is relative humidity in the flow and RH_{bed} is relative humidity in sample bed.

S8. Gas sorption measurements

Before performing the gas sorption experiments, a freshly prepared sample of $[Zn_3(OH)_2(btca)_2]$ was placed in a quartz tube and degassed under high vacuum using a Smart VacPrep instrument at 393K for 24 h to remove any remaining solvent molecules. Isotherms were measured using a Micromeritics 3Flex sorption analyser. Gases were used as obtained from BOC Gases (Ireland), with the following certified purities: research-grade CO_2 (99.995%). Bath temperature of 195 K were maintained using dry ice–acetone slurry. Sample was activated between successive experiments overnight or for a minimum of 5 h at 393 K under high vacuum.

S9. Analysis of previous guest-induced transformations in $[Zn_3(OH)_2(btca)_2]$ for hydrated and DMF included crystal structures from the Cambridge Structural Database (CSD)

Cabrero-Antonino and co-workers[8] synthesized $[Zn_3(OH)_2(btca)_2]\cdot 2DMF\cdot 1H_2O$ (CSD identifier BORLUS) and carried out desolvation experiments to track the solvent-induced transformations that occur due to solvent loss. Following equilibration at room temperature, which resulted in the removal of the included water to afford $[Zn_3(OH)_2(btca)_2]\cdot 2DMF$ (CSD identifier BORLEC), the crystals were exposed to a target temperature *T* at which the crystals were kept for 2 h followed by SCXRD analysis at 426K. The non-ambient temperatures *T* that were evaluated were 373K (CSD identifier BORLIG), 473K (CSD identifier MUGHED01) and 623K (CSD identifier BORLOM). Their TGA analysis shows that heating the as-synthesized crystals above 573K would result in the loss of 20 wt% which is equivalent to all of the included solvent molecules. However, the crystal structure determined after heating to 623K is in the phase NP and has the formula $[Zn_3(OH)_2(btca)_2]\cdot 4H_2O$ indicating that a stoichiometric number of water molecules were included within its structure. From our analyses, exposing activated crystals to the atmosphere results in the conversion LP-β→NP**.** Therefore, it is probable that heating the crystal to 623K resulting in a conversion to the phase LP-β, and that the crystal adsorbed atmospheric moisture during handling affordingNP.

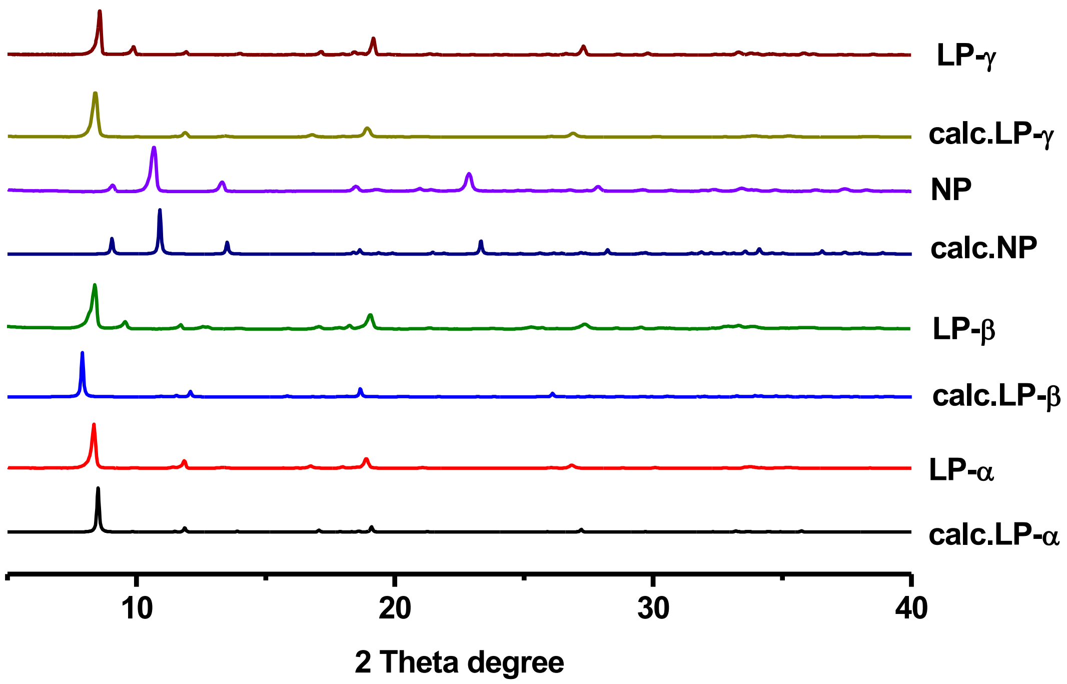

Figure S1: Comparison of calculated [5] and experimental PXRD diffractograms of $[Zn_3(OH)_2(btca)_2]$.

Table S1: Selected crystallographic details for the guest-free and guest loaded for of $[Zn_3(OH)_2(btca)_2]$

Unit Cell Parameters	LP-β	NP	LP-γ	Reported LP-α[1]
Formula	$[Zn_3(OH)_2(btca)_2]$	$[Zn_3(OH)_2(btca)_2]\cdot 2H_2O$	$[Zn_3(OH)_2(btca)_2]\cdot 4H_2O$	$[Zn_3(OH)_2(btca)2]\cdot$DMF$\cdot 4H_2O$
Formula weight (g/mol)	555	591	627	700
Temperature (K)	298K	100K	100K	298K
Crystal system	Monoclinic	Monoclinic	Monoclinic	Monoclinic

Space group	C2/c	C2/c	C2/c	C2/c
a (Å)	17.7713(6)	19.537(4)	18.5505(7)	17.9856(4)
b (Å)	12.9777(5)	8.9132(19)	11.6548(5)	12.7314(4)
c (Å)	10.9444(3)	11.096(2)	11.0031(4)	11.0892(3)
α (°)	90	90	90	90
β (°)	93.2330(10)	90.164(9)	91.6970(10)	93.206(2)
γ (°)	90	90	90	90
V ($Å^3$)	2520.10(15)	1932.22	2377.85	2535.25
Z	4	4	4	4
GooF	1.056	1.111	1.063	1.106
R_1 [$I > 2\sigma(I)$]	4.46	7.10	3.30	3.19
WR_2 [all data]	12.28	18.43	7.75	8.91

Table S2: O···O distances (Å) for possible hydrogen bonds involving water molecules modelled for LP-γ at −173 °C.

	O3	O14	O15	O1WA	O2WA	O3WA	O4WA
O1WA	2.830				2.947	3.004	2.655
O2WA		3.010				2.661	2.439
O3WA			3.183			2.547	
O4WA							

Symmetry operators $1: 1/2-x,1/2-y,1-z; $2: 1/2-x,1/2+y,1.5-z; S3: 1-x,-y,1-z; $4: 1-x,y,1.5-z; $5: 1-x,1+y,1.5-z

Table S3: O···O distances (Å) for possible hydrogen bonds involving water molecules modelled for NP at −173 °C.

	O3	O14	O15	O1WA	O2WA
O1WA	2.740				2.741
O2WA		2.839	3.073		3.133

Symmetry operators $1: 1/2+x,1/2+y,z; $2: -x,y,1.5-z; $3: -x,1-y,1-z; $4: 1-x,1-y,1-z;

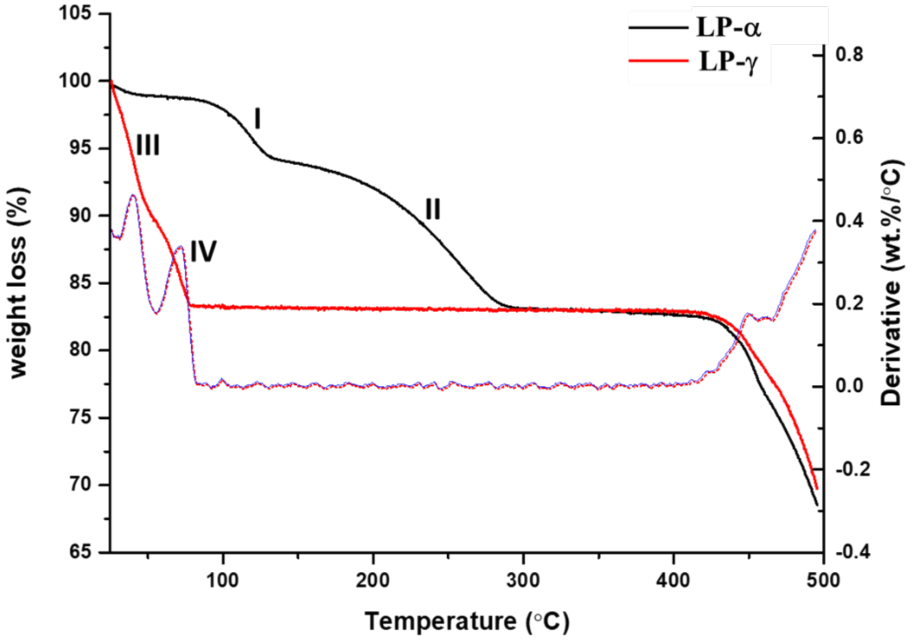

Figure S2: TGA curves of LP-**α** (black) and LP-γ (red) under N_2 flow and the corresponding guest lost during heating: I, 4 H_2O; II, DMF; III, 4 H_2O; IV, 4 H_2O. The derivative (wt%/°C) of LP-γ is shown as a blue line.

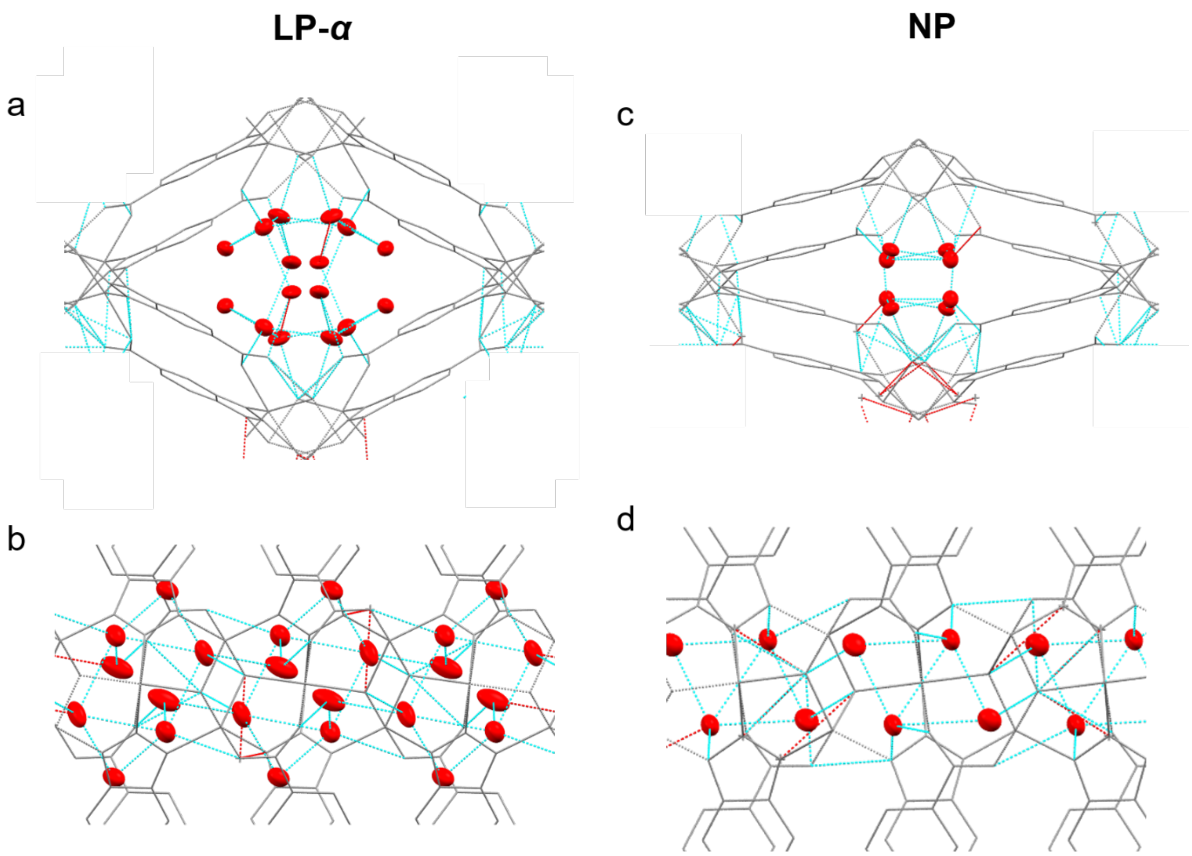

Figure S3: Projections of LP-*α* along (a) [001] and (b) [010], and NP along (c) [001] and (d) [010] showing the hydrogen-bonded network of water molecules in host frameworks (wireframe, grey) channels. Water oxygen atoms (red) of the major disordered components (atoms OW1A-4A for LP-*α* and OW1A-2A for NP) are shown as ellipsoids (50% probability level) corresponding to their anisotropic displacement parameters. Interatomic O_{H2O}-O_{H2O} and O_{H2O}-$O_{framework}$ distances (blue dashed lines) shown range between 2.4 and 3.2 Å. Some atoms were omitted for clarity.

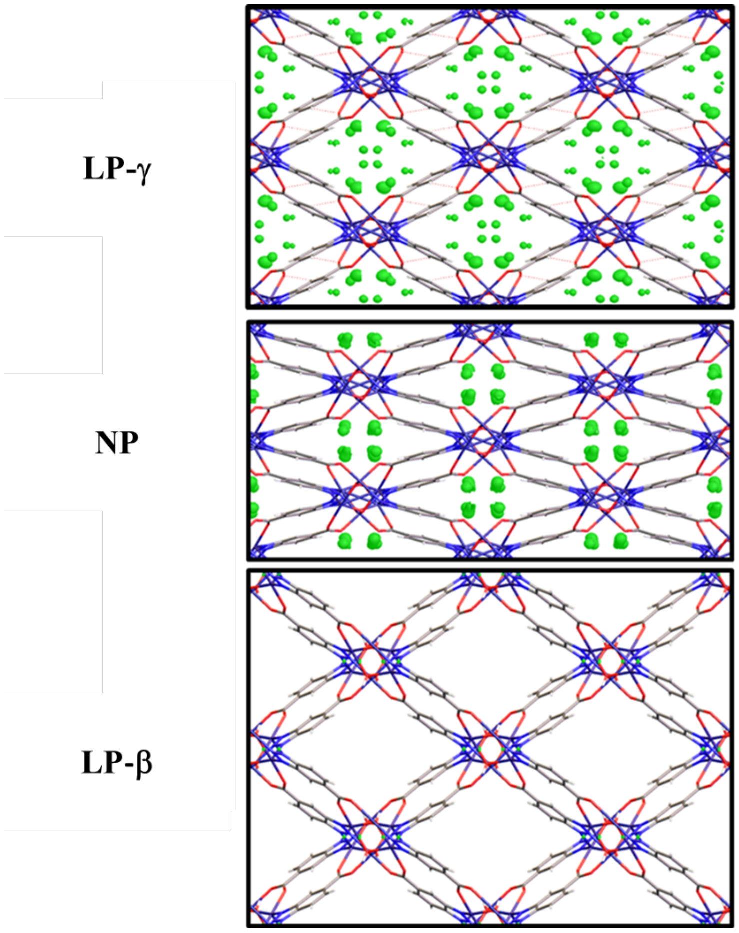

Figure S4: Difference electron density maps (green) calculated from refined crystal structures (Table S2) of LP-γ (3 e^- $Å^{-3}$), NP (3 e^- $Å^{-3}$) and LP-β (1 e^- $Å^{-3}$) using OLEX2.[9] Colors: carbon, grey; nitrogen, blue; oxygen, red; hydrogen, white.

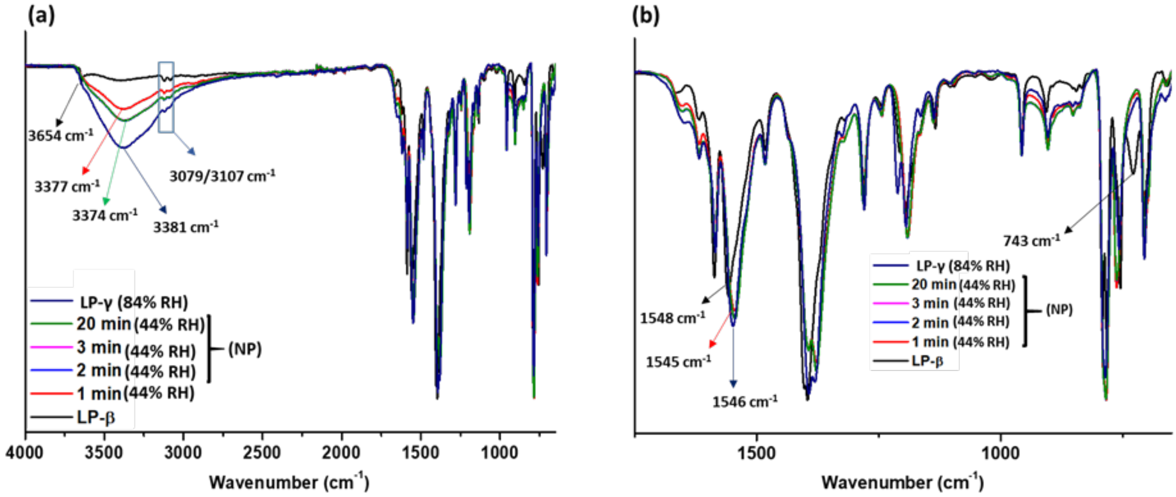

Figure S5: Fourier transform infrared spectroscopy (FT-IR) spectra measured for LP-β, NP and LP-γ showing the (a) full range and (b) the fingerprint region. The time values indicate the time elapsed after first exposure.

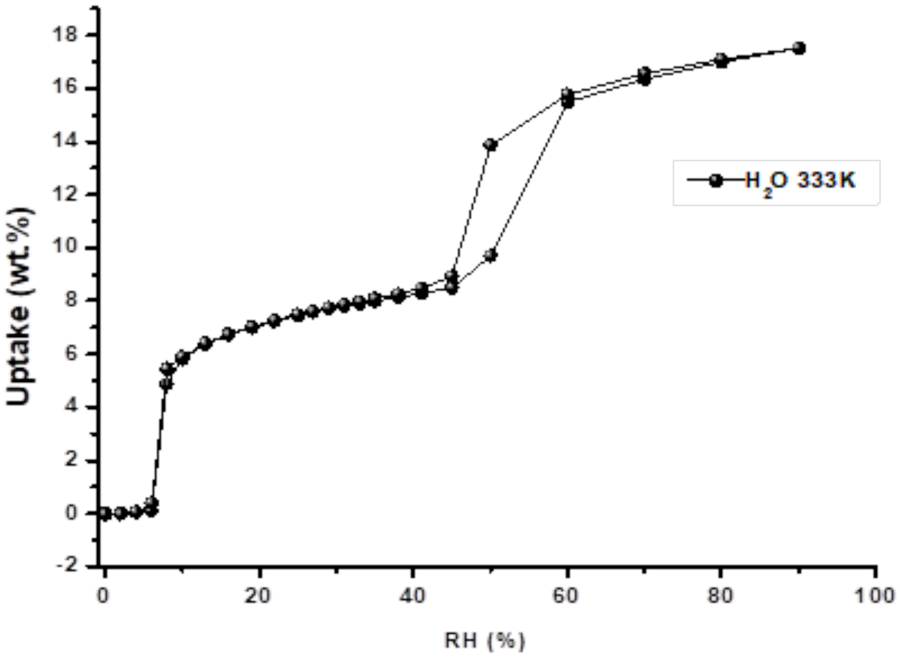

Figure S6: Water vapor sorption isotherm of [$Zn_3(OH)_2(btca)_2$] collected using an dynamic vapor sorption (DVS) instrument at 333K.

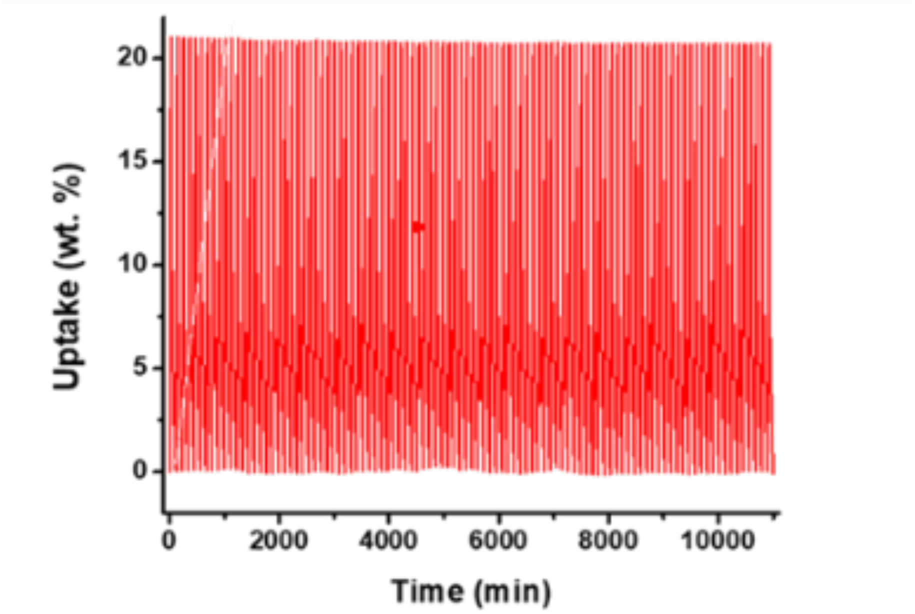

Figure S7: Water vapor humidity swing cycling of [$Zn_3(OH)_2(btca)_2$]   (0% RH to 60% RH).

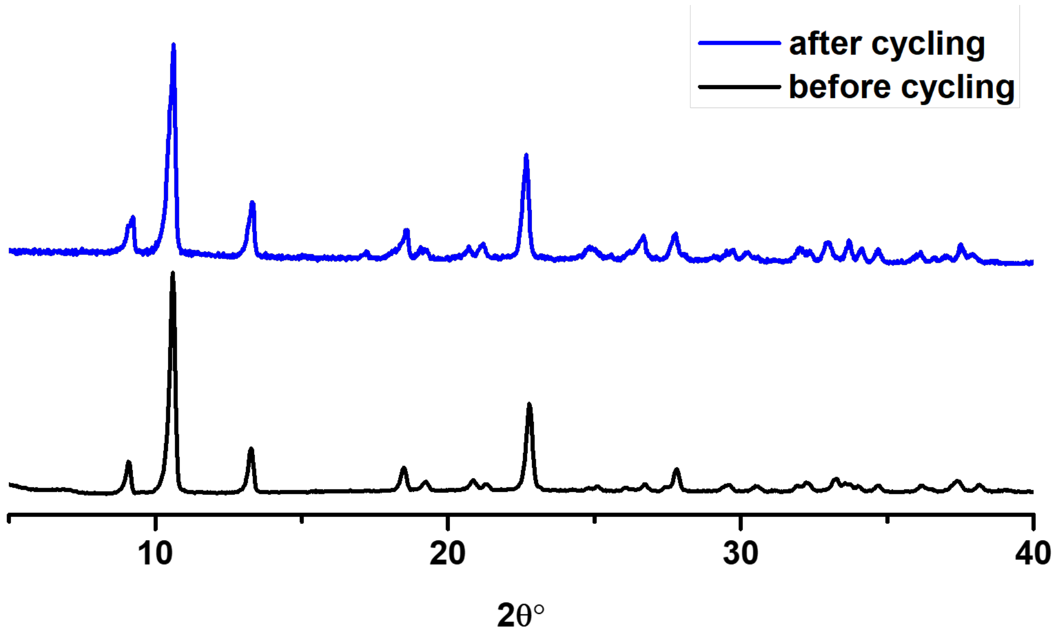

Figure S8: Comparison of PXRD pattern before and after 100 regeneration cycles.

Tables S4: Comparison of literature reports for materials exhibiting multiple steps in the water vapor adsorption isotherm. The RH value (%), maximum uptake (wt.%) of each step and application is reported.

Sorbent	RMOM or FMOM	1ST step		2ND step		3RD step		Application	Reference
		RH (%)	Uptake	RH (%)	Uptake	RH (%)	Uptake		
**MIL-100(Fe)**	RMOM	30	590^b	45	1000^b	-	-	AWH	10
**MIL-101 (Cr)**	RMOM	43	0.75^c	50	1.3^c	-	-	IHC	11
**MIL-101-NH$_2$**	RMOM	36	0.6^c	43	0.9^c	-	-	IHC	11
**MIL-101-SO$_3$H**	RMOM	27	0.4^c	35	0.7^c	-	-	AWH	11
**[Mn(imH)]$_2$[Mo(CN)$_8$]**	FMOM	18	6^a	21	13^a	30%	25^a	AWH	12
**[FeII(prentrz)$_2$ PdII(CN)$_4$]**	FMOM	8	1.4^d	70	2.7^d	-	-	AWH	13
**MIL-53(Cr)**	FMOM	12	0.07^c	100	0.38^c	-	-	AWH	14
**[Zn$_3$(OH)$_2$(btca)$_2$]**	FMOM	5	12^a	47	25^a	-	-	AWH & IHC	This work

^{a}wt%, ^{b}cm^3 g^{-1}, ^{c}g g^{-1}, ^{d}per Fe atom

.

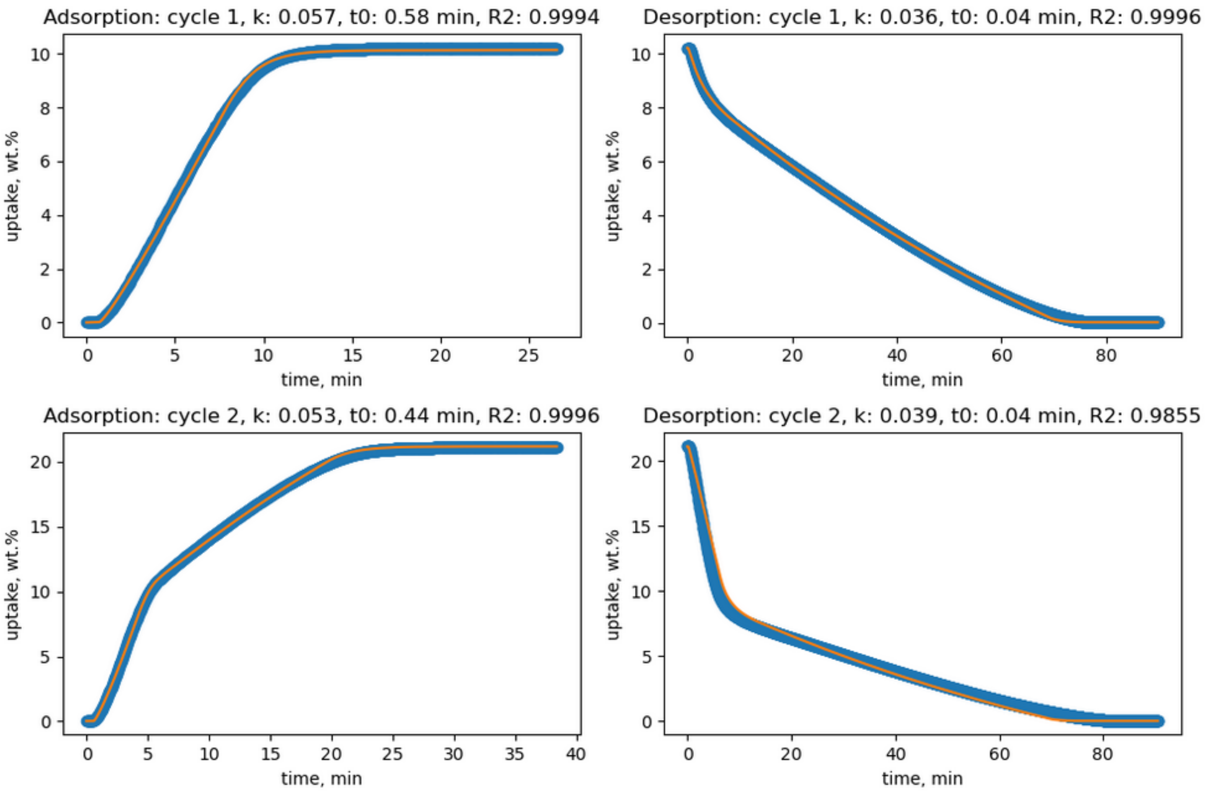

Figure S9: Fit of kinetics data (blue) at 300K on [$Zn_3(OH)_2(btca)_2$] using isotherm-based kinetics model[7] (orange) and corresponding fit parameters for 0-30 % RH (**top**) and 0-60 % RH (**bottom**) humidity swing.

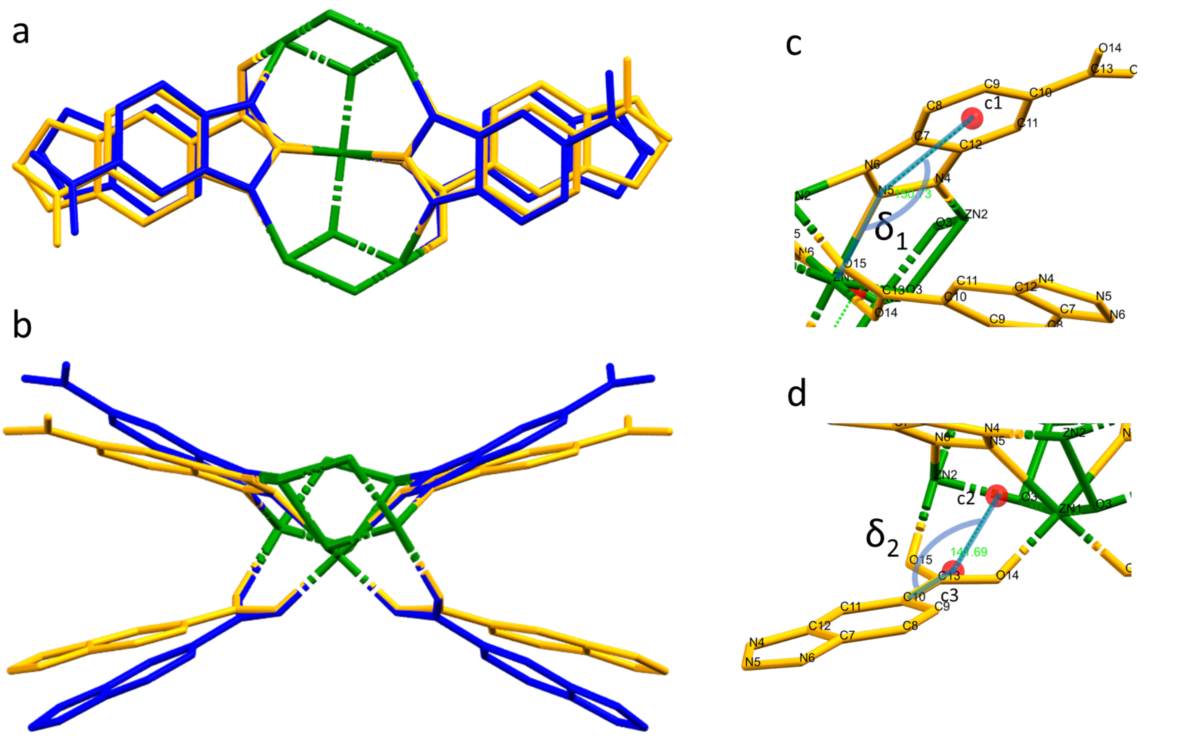

Figure S10: Structural overlays of LP-γ (blue) and NP (yellow) with overlayed Zn and O atoms from both crystal structures shown in green generated using Mercury.[5] Projections along the (a) *b* and (b) *c* axes. (c,d) Centroids (red spheres) c1 (C8 and C11), c2 (Zn1 and Zn2) and c3 (O14 and O15) were used to calculated the angles δ_1 (C1-N5-Zn1) and δ_2 (c2-c3-C10) shown in blue. Hydrogen atoms were omitted for clarity.

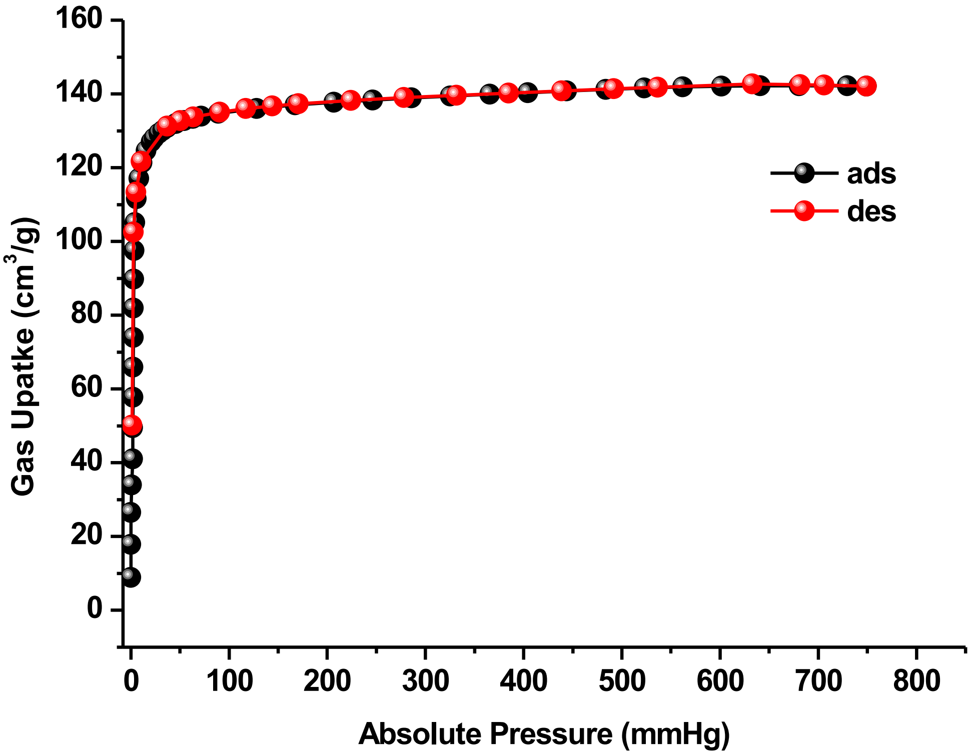

Figure S11: Equilibrium CO_2 adsorption and desorption isotherms measured for LP-β at 195 K.

Table S5: Reported crystal structures of [$Zn_3(OH)_2(btca)_2$] with selected details retrieved from the CSD: initial phase, treatment before SCXRD experiment, guest molecules modelled, phase determined by SXCRD and whether sorption analysis was carried out.

Refcode	Guest(s) (Host:Guest)	Initial Phase	Treatment	Phase determined by SCXRD	Sorption	Reference
**BORLUS**	DMF/H_2O	**α·2DMF·H_2O**	As-synthesized	**α·2DMF·H_2O**		
**BORLEC**	DMF	**α·2DMF·H_2O**	RT for 24 h			
**BORLIG**	DMF	**α·2DMF·H_2O**	100 °C for 2 h		None	[8]
**MUGHED01**	DMF	**α·2DMF·H_2O**	200 °C for 2 h			
**BORLOM**	H_2O	**α·2DMF·H_2O**	350 °C for 2 h	**β·4H_2O**		
**FIWXUH**	Acetylene	**α·DMF·4H_2O**	C_2H_2 atmosphere at RT for 6 h	**β·C_2H_2**	C_2H_2 in situ	[15]
**FIWYUI**	Guest free	**α·DMF·4H_2O**	200 °C dynamic vacuum for 36 h	**γ**	CO_2,C_2H_2, C_2H_4, C_2H_6	[15]
**LOPJIM**	CO_2	**α·DMF·4H_2O**	CO_2 atmosphere at RT for 6 h	**β·CO_2**	CO_2 in situ	[15]
**LETRIN**	DMF/H_2O	**α·DMF·4H_2O**	As-synthesized	**α·DMF·4H_2O**	CO_2,N_2	[1]
**LETROT**	DMF/0.5H_2O	**α·DMF·4H_2O**	220 °C for 24 h in air	**α·DMF·0.5H_2O**	None	[1]

**MUGHED01**	DMF	***α*·DMF·4H_2O**	150 °C for 2 h	***α*·DMF**	High pressure CO_2, N_2	[16]
**LETRUZ**	H_2O	***α*·DMF·0.5H_2O**	220 °C for 48 h in DMF	***β*·2H_2O**	None	[1]

Computational methodology

Periodic Density Functional Theory (DFT) calculations were performed using the projected augmented wave (PAW) formalism[17] as implemented in the Vienna Ab Initio Simulation Package (VASP 5.4.4),[18, 19] employing the BEEF-vdW exchange-correlation functional.[20] The atomic positions in the structures of [$Zn_3(OH)_2(btca)_2$] (14 different configurations or polymorphs) were optimized at their experimentally refined cell parameters (**Table S6**), using the conjugate gradient algorithm with force and electronic convergence criteria of 0.02 eV/Å and 10^{-5} eV, respectively, a Gaussian smearing of 0.05 eV, an energy cutoff of 500 eV[21]. Using the Nudged Elastic Band (NEB) as implemented in VASP5.4.4, two NEB runs with each 8 intermediary images were performed at the Γ-point. The first NEB run considered the 8 intermediary structures between the NP of [$Zn_3(OH)_2(btca)_2$]·4H_2O (a = 19.569 Å, b = 8.9227 Å, c = 11.109, α = γ = 90º, β = 90.122º) and a LP-γ of [$Zn_3(OH)_2(btca)_2$]·8H_2O (a = 18.312 Å, b = 12.077 Å, c = 11.014, α = γ = 90º, β = 91.885º). The second NEB run considered the transition from the latter to an arbitrary determined overstretched open phase with larger unit cell volume (V = 2669.51 $Å^3$; a = 16.538 Å, b = 15.322 Å, c = 10.646, α = γ = 90º, β = 97.387º). The unit cell parameters for the intermediate images in these NEB runs were found via linear interpolation and were kept fixed, while the default spring constant of 5 eV/$Å^2$ was employed between the images. The structures were ranked according to increasing volume and fourteen structures were selected for subsequent grand canonical Monte Carlo (GCMC) simulations to study the H_2O adsorption isotherms. The structures were ranked according to increasing volume (see **Table S6**) and geometry optimization using a 3x3x3 Monkhorst-Pack[21] k-point grid.

Table S6: Unit cell parameters of the 14 simulated crystal structures employed in NEB-run 1 and NEB-run 2 to simulated water sorption isotherms (labeled 00, 01, …, 13). The experimental crystal structures of NP, LP-γ and LP-β correspond most closely to the simulated crystal structures 00, 09 and 10, respectively.

	NEB-run	a (Å)	b (Å)	c (Å)	α (º)	β (º)	γ (º)	V ($Å^3$)	Exp.	Rel. Energy (kJ/mol)
00	1	19.569	8.9227	11.109	90	90.122	90	1939.72	NP	0.0
01	1	19.429	9.273	11.098	90	90.316	90	1999.62		
02	1	19.289	9.623	11.088	90	90.511	90	2058.32		
03	1	19.150	9.974	11.078	90	90.706	90	2115.82		
04	1	19.010	10.324	11.067	90	90.902	90	2172.13		
05	1	18.870	10.675	11.058	90	91.097	90	2227.24		
06	1	18.731	11.025	11.048	90	91.294	90	2281.15		
07	1	18.591	11.376	11.038	90	91.490	90	2333.88		
08	1	18.451	11.726	11.029	90	91.687	90	2385.43		
09	1 & 2	18.312	12.077	11.019	90	91.885	90	2435.79	LP-γ	-32.5
	2	18.112	12.437	10.976	90	92.554	90	2470.30		
10	2	17.913	12.798	10.9333	90	93.233	90	2502.67	LP-β	-27.5
	2	17.715	13.159	10.890	90	93.921	90	2532.88		
11	2	17.517	13.519	10.848	90	94.620	90	2560.97		
	2	17.320	13.880	10.807	90	95.329	90	2586.92		
12	2	17.123	14.240	10.766	90	96.048	90	2610.74		
	2	16.927	14.601	10.725	90	96.779	90	2632.44		
	2	16.731	14.961	10.685	90	97.520	90	2652.03		
13	2	16.537	15.322	10.645	90	98.275	90	2669.51		

Grand Canonical Monte Carlo (GCMC) simulations were performed to achieve more insight into the H_2O adsorption and breathing trajectory at 298.15 K in (1x1x2) supercell of [$Zn_3(OH)_2(btca)_2$]. For the GCMC simulations, the unit cells and framework atoms were kept fixed at their DFT-optimized

positions. The adsorption isotherms were calculated for the pressure range of 0 to 64 kPa. Then we plotted the water uptake versus the scaled relative humidity RH*% = c× RH%, in which the coefficient was chosen as c = 0.05. This scaling was necessary so that the simulated uptake was lower or equal to the experimental uptake at high RH*-%. Furthermore, we are well aware that the employed water models lack interaction terms (*e.g.* polarization), which automatically results in an underestimation of the adsorption interactions. As state-of-the-art force fields are not able to describe water and its interactions, this justifies a 'scaling' of computational data for the sole purpose of gaining insight into unit cell volume variations as function of the relative humidity (RH-%).

The point charges were calculated via the charge equilibration (Qeq) method[22] followed by GCMC simulations in Material Studio employing the COMPASS II force field[23]. The frameworks point charges are supplied in Supplementary CIF-files. The water molecule's point charges [e] are presented in **Figure S12**.

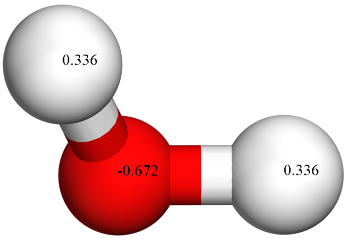

Figure S12: Water molecule model with atomic point charges according to the Qeq method.

In the grand canonical ensemble, the Metropolis sampling method considered different moves, such as translation (corresponds to translation of the center-of-mass of the selected adsorbate molecule), rotation (rotating the selected adsorbate molecule), regrowth (removing a selected adsorbate molecule from the system and reintroducing it at a random position with random orientation), and conformer (collecting multiple sorbate conformations), with relative probabilities of 1, 1, 0.1 and 1, respectively. We applied a cut off distance of 7 Å and truncated the potential using a cubic spline with a spline width of 1 Å. Each GCMC simulation included 5×10^6 equilibration steps, followed by 5×10^6 production steps to ensure reasonable ensemble averages.[23] **Figure S13** shows the adsorption isotherms simulated using GCMC in fourteen structures of different cell volumes (**Table S6**). It is clear that the experimentally observed isotherms of H_2O adsorption cannot be associated with a single isotherm simulated assuming rigidity of the host framework and that the abrupt increase of the adsorbed amount is a consequence of a structural transition within the adsorbent leading to a change of accessible volume.[24] For the simulated adsorption isotherms, the shape of the curves changes from Langmuir adsorption isotherm to an S-shaped isotherm when increasing the cell volume (**Figure S13**). Langmuir adsorption isotherms typically represent adsorption in a unimolecular layer,[25] which was observed for the systems with unit cell volumes between 1939.72 and 2281.74 Å^3 (systems with codes 0 to 6 in Table S6). By further increasing the unit cell volume from 2334.67 to 2669.54 Å^3 (*e.g.* system codes 8 to 13, (**Table S6**)), the shape of the simulated isotherms resembled better S-shaped isotherms (**Figure S13**), representing unrestricted monolayer-multilayer adsorption (**Figure S13**).Hydrogen bond interactions based on selected structures could be followed based on the adsorption process (**Figure S14**)

To get more insight into the framework induced fit mechanism during H_2O adsorption process and the water loading in the framework, a 3-dimensional adsorption process representation was constructed; water uptake versus RH* %, and unit cell volume (see **Figure S15**). By indicating the experimental

water sorption isotherm on this 3-dimensional representation, we can predict a plausible unit cell volume at every point of the experimental isotherms and, thus derive the framework induced fit behavior (see **Figure S15**).

Furthermore, the adsorption energy was determined after optimizing selected structures from GCMC-simulations, and subsequently decomposed into water-host, water-water and framework deformation energies per water molecule; 1x1x2 supercell structures of [$Zn_3(OH)_2(btca)_2$] with varying amount of water were optimized using the BEEFvdW functional (**Table S7**).

Table S7: Water adsorption energy per water (E_{ads}) as function of loading in 1x1x2 supercells determined after optimization of the relevant GCMC-outcome structures using BEEFvdW functional. The energy decomposition ($E_{host-guest}$, $E_{guest-guest}$, $E_{host\_deformation}$) allows the quantification of the different interactions per adsorbed water molecule, i.e. host-guest, guest-guest, framework-deformation energy, respectively.

	RH*-%.	H_2O loading	E_{ads} (kJ/mol)	$E_{host-guest}$ (kJ/mol)	$E_{guest-guest}$ (kJ/mol)	$E_{host\_deformation}$ (kJ/mol)	α (°)	β (°)	γ (°)	V (Å³)	Exp.
03	2.52	8	-56.7	-46.0	-13.5	2.7	90	90.706	90	2115.82	
04	31.55	11	-59.9	-45.3	-17.9	3.3	90	90.902	90	2172.13	
09	44.16	21	-52.5	-25.8	-28.2	1.4	90	91.885	90	2435.79	LP-γ
11	50.47	28	-51.1	-24.7	-28.5	2.1	90	94.620	90	2560.97	

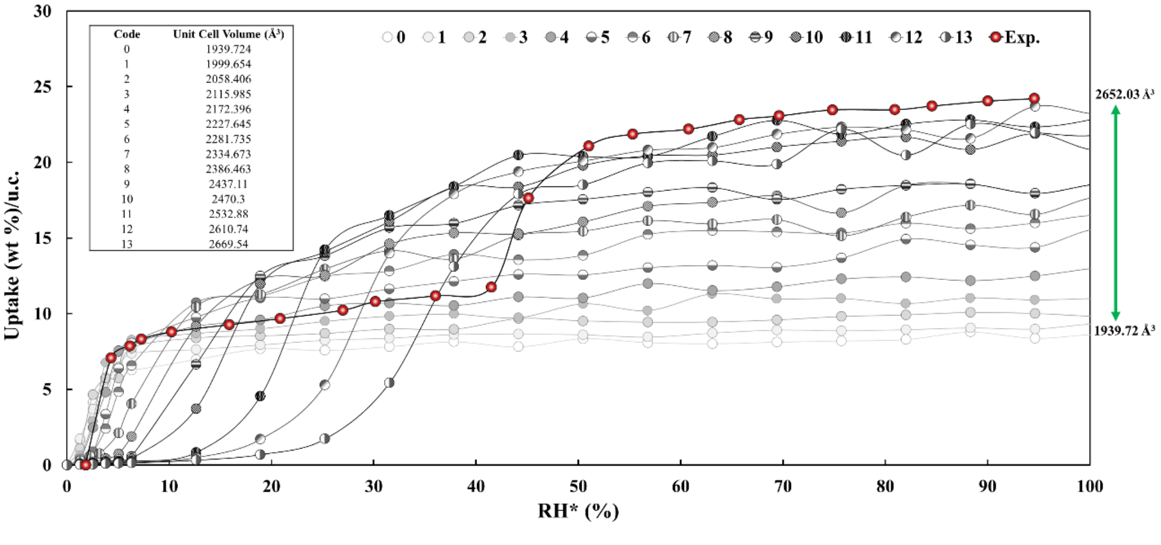

Figure S13: Rescaled simulated adsorption isotherms of H_2O at different unit cell volumes [$Zn_3(OH)_2(btca)_2$], so that the simulated maximum adsorption (in wt%) falls below the experimental data. Each isotherm was calculated at the fixed unit cell volume. The codes represent the cells with different unit cell volumes. The red points represent the experimental data.

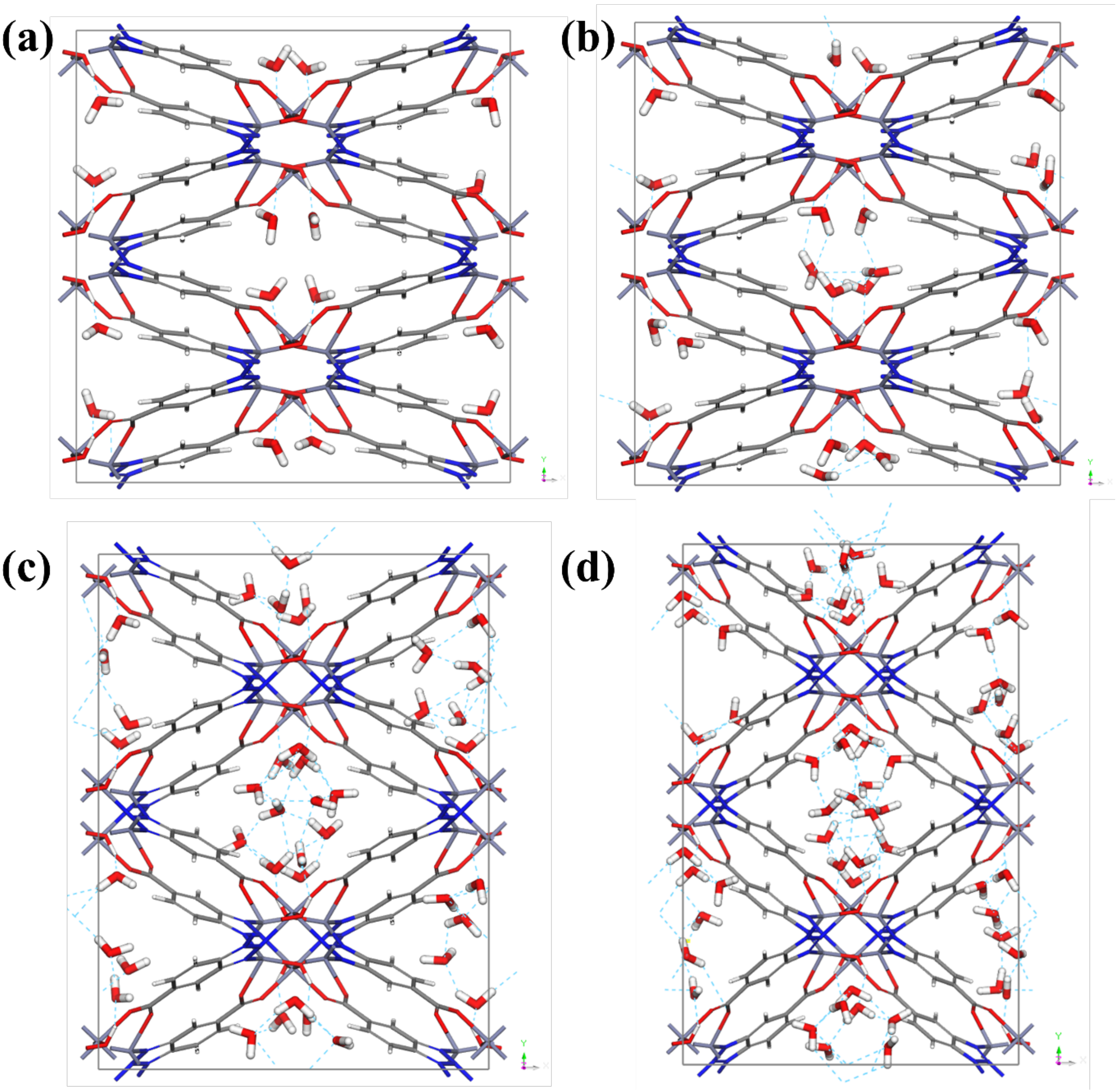

Figure S14: Visualization of hydrogen bonds (presented in dashed blue lines) for selected structures from **Table S7**, (a) 03, (b) 04, (c) 09, and (d) 11.

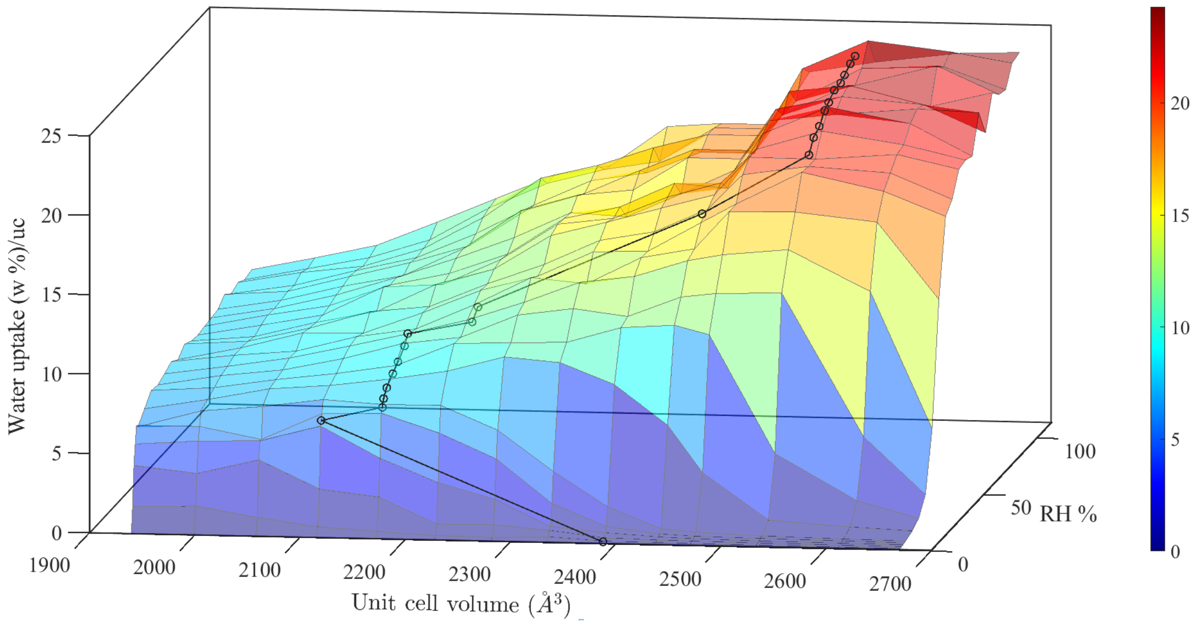

Figure S15: 3-dimensional H_2O adsorption isotherms taken from GCMC. The black circles/lines represent the predictive $[Zn_3(OH)_2(btca)_2]$ induced fit mechanism during H_2O adsorption process.

References

(1) Xiao, J.; Wu, Y.; Li, M.; Liu, B.-Y.; Huang, X.-C.; Li, D. Crystalline Structural Intermediates of a Breathing Metal-Organic Framework That Functions as a Luminescent Sensor and Gas Reservoir. *Chem. Eur. J.* **2013**, *19* (6), 1891-1895. DOI: 10.1002/chem.201203515.

(2) Francart, T.; van Wieringen, A.; Wouters, J. APEX 3: a multi-purpose test platform for auditory psychophysical experiments. *J.Neurosci.Meth.* **2008**, *172* (2), 283-293. DOI: 10.1016/j.jneumeth.2008.04.020.

(3) Moriguchi, T.; Egami, T.; Yakeya, D.; Jalli, V.; Yoza, K.; Tsuge, A. Synthesis and Crystal Structure Determination of 4’,9’,4”,9”-Tetra- t -Butyl- 1’,6’,1”,6”-Tetramethoxy-2,5-Dithia[3.3] Metabiphenylophane. *Crystal Structure Theory and Applications* **2016**, *05* (03), 56-62. DOI: 10.4236/csta.2016.53005.

(4) Sheldrick, G. M. Crystal structure refinement with<i>SHELXL</i>. *Acta Crystallographica Section C Structural Chemistry* **2015**, *71* (1), 3-8. DOI: 10.1107/s2053229614024218.

(5) Macrae, C. F.; Sovago, I.; Cottrell, S. J.; Galek, P. T. A.; McCabe, P.; Pidcock, E.; Platings, M.; Shields, G. P.; Stevens, J. S.; Towler, M.; et al. Mercury 4.0: from visualization to analysis, design and prediction. *Journal of Applied Crystallography* **2020**, *53*, 226-235. DOI: 10.1107/S1600576719014092.

(6) Dolomanov, O. V.; Bourhis, L. J.; Gildea, R. J.; Howard, J. A. K.; Puschmann, H. <i>OLEX2</i>: a complete structure solution, refinement and analysis program. *Journal of Applied Crystallography* **2009**, *42* (2), 339-341. DOI: 10.1107/s0021889808042726.

(7) Bezrukov, A. A.; O'Hearn, D. J.; Gascon-Perez, V.; Darwish, S.; Kumar, A.; Sanda, S.; Kumar, N.; Francis, K.; Zaworotko, M. J. Metal-organic frameworks as regeneration optimized sorbents for atmospheric water harvesting. *Cell Rep.Phys.Sci* **2023**, *4* (2). DOI: ARTN 10125210.1016/j.xcrp.2023.101252.

(8) Cabrero-Antonino, M.; Remiro-Buenamañana, S.; Souto, M.; García-Valdivia, A. A.; Choquesillo-Lazarte, D.; Navalón, S.; Rodríguez-Diéguez, A.; Mínguez Espallargas, G.; García, H. Design of cost-efficient and photocatalytically active Zn-based MOFs decorated with Cu₂O

nanoparticles for CO₂methanation. *Chem. Commun.* **2019**, *55* (73), 10932-10935. DOI: 10.1039/c9cc04446a.
(9) Dolomanov, O. V.; Bourhis, L. J.; Gildea, R. J.; Howard, J. A. K.; Puschmann, H. OLEX2: a complete structure solution, refinement and analysis program. *Journal of Applied Crystallography* **2009**, *42*, 339-341. DOI: 10.1107/S0021889808042726.
(10) Küsgens, P.; Rose, M.; Senkovska, I.; Fröde, H.; Henschel, A.; Siegle, S.; Kaskel, S. Characterization of metal-organic frameworks by water adsorption. *Microporous Mesoporous Mater.* **2009**, *120* (3), 325-330. DOI: https://doi.org/10.1016/j.micromeso.2008.11.020.
(11) Akiyama, G.; Matsuda, R.; Sato, H.; Hori, A.; Takata, M.; Kitagawa, S. Effect of functional groups in MIL-101 on water sorption behavior. *Microporous Mesoporous Mater.* **2012**, *157*, 89-93. DOI: 10.1016/j.micromeso.2012.01.015.
(12) Magott, M.; Gaweł, B.; Sarewicz, M.; Reczyński, M.; Ogorzały, K.; Makowski, W.; Pinkowicz, D. Large breathing effect induced by water sorption in a remarkably stable nonporous cyanide-bridged coordination polymer. *Chem. Sci.* **2021**, *12* (26), 9176-9188. DOI: 10.1039/d1sc02060a.
(13) Xue, J.-P.; Hu, Y.; Zhao, B.; Liu, Z.-K.; Xie, J.; Yao, Z.-S.; Tao, J. A spin-crossover framework endowed with pore-adjustable behavior by slow structural dynamics. *Nat.Commun.* **2022**, *13* (1). DOI: 10.1038/s41467-022-31274-8.
(14) Bourrelly, S.; Moulin, B.; Rivera, A.; Maurin, G.; Devautour-Vino, S.; Serre, C.; Devic, T.; Horcajada, P.; Vimont, A.; Clet, G.; et al. Explanation of the Adsorption of Polar Vapors in the Highly Flexible Metal Organic Framework MIL-53(Cr). *J. Am. Chem. Soc* **2010**, *132* (27), 9488-9498. DOI: 10.1021/ja1023282.
(15) Zeng, H.; Xie, M.; Huang, Y. L.; Zhao, Y. F.; Xie, X. J.; Bai, J. P.; Wan, M. Y.; Krishna, R.; Lu, W. G.; Li, D. Induced Fit of C2H2 in a Flexible MOF Through Cooperative Action of Open Metal Sites. *Angewandte Chemie-International Edition* **2019**, *58* (25), 8515-8519. DOI: 10.1002/anie.201904160.
(16) Yue, Y.; Rabone, J. A.; Liu, H.; Mahurin, S. M.; Li, M.-R.; Wang, H.; Lu, Z.; Chen, B.; Wang, J.; Fang, Y.; et al. A Flexible Metal–Organic Framework: Guest Molecules Controlled Dynamic Gas Adsorption. *J. Phys. Chem.C* **2015**, *119* (17), 9442-9449. DOI: 10.1021/acs.jpcc.5b02359.
(17) Blöchl, P. E. Projector augmented-wave method. *Physical Review B* **1994**, *50* (24), 17953-17979. DOI: 10.1103/physrevb.50.17953.
(18) Kresse, G.; Furthmüller, J. Efficiency of ab-initio total energy calculations for metals and semiconductors using a plane-wave basis set. *Computational Materials Science* **1996**, *6* (1), 15-50. DOI: https://doi.org/10.1016/0927-0256(96)00008-0.
(19) Kresse, G.; Furthmüller, J. Efficient iterative schemes for<i>ab initio</i>total-energy calculations using a plane-wave basis set. *Physical Review B* **1996**, *54* (16), 11169-11186. DOI: 10.1103/physrevb.54.11169.
(20) Wellendorff, J.; Lundgaard, K. T.; Møgelhøj, A.; Petzold, V.; Landis, D. D.; Nørskov, J. K.; Bligaard, T.; Jacobsen, K. W. Density functionals for surface science: Exchange-correlation model development with Bayesian error estimation. *Physical Review B* **2012**, *85* (23). DOI: 10.1103/physrevb.85.235149.
(21) Monkhorst, H. J.; Pack, J. D. Special points for Brillouin-zone integrations. *Physical Review B* **1976**, *13* (12), 5188-5192. DOI: 10.1103/physrevb.13.5188.
(22) Lucas, T. R.; Bauer, B. A.; Patel, S. Charge equilibration force fields for molecular dynamics simulations of lipids, bilayers, and integral membrane protein systems. *Bba-Biomembranes* **2012**, *1818* (2), 318-329. DOI: 10.1016/j.bbamem.2011.09.016.
(23) BIOVIA, Dassault Systèmes, Materials Studio, San Diego: Dassault Systèmes, 2022.
(24) Formalik, F.; Neimark, A. V.; Rogacka, J.; Firlej, L.; Kuchta, B. Corrigendum to "Pore opening and breathing transitions in metal-organic frameworks: Coupling adsorption and deformation" [J. Colloid Interface Sci. 578 (2020) 77–88]. *J Colloid Interf Sci* **2022**, *611*, 771. DOI: https://doi.org/10.1016/j.jcis.2021.12.112.
(25) Brunauer, S.; Deming, L. S.; Deming, W. E.; Teller, E. On a Theory of the van der Waals Adsorption of Gases. *J. Am. Chem. Soc* **1940**, *62* (7), 1723-1732. DOI: 10.1021/ja01864a025.
